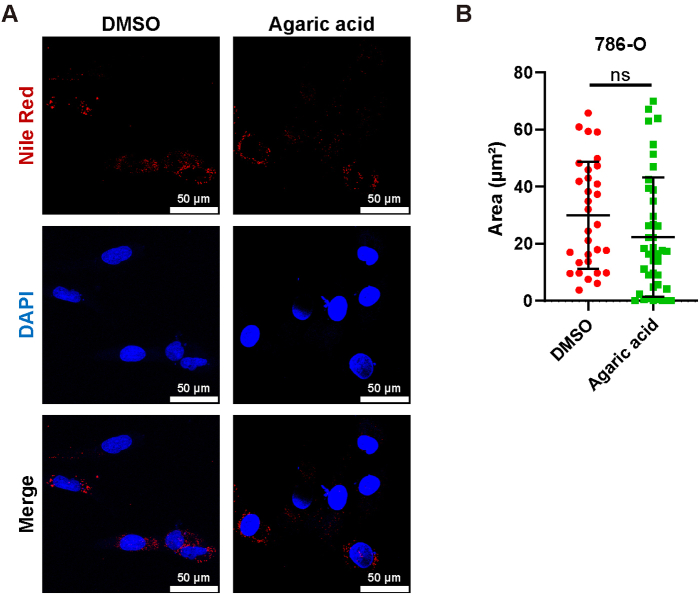# The mediator subunit complex protein MED15 promotes lipid deposition and cancer progression during hypoxia

**DOI:** 10.1016/j.jbc.2025.108296

**Published:** 2025-02-11

**Authors:** Boqi Zhang, Yu Zhu, Yanfei Tang, Lu Liu, Yunzhang Liu, Yun Li, Wengong Yu, Ling Lu

**Affiliations:** 1 Key Laboratory of Marine Drugs, The Ministry of Education of China, School of Medicine and Pharmacy, Ocean University of China, Qingdao, China; 2 Laboratory for Marine Drugs and Bioproducts, Qingdao Marine Science and Technology Center, Qingdao, China

**Keywords:** MED15, HIF, CPT1A, cancer, hypoxia

## Abstract

Hypoxia, a hallmark of solid tumors, is associated with increased lipid droplet (LD) accumulation. However, the mechanisms underlying this remain elusive. Here, we identify Mediator complex subunit 15 (MED15) as a critical regulator of hypoxia-inducible factor (HIF) signaling, potentially impacting LD accumulation. In mammalian cells, we elucidated that MED15, as a HIF target gene, participates in promoting HIF transcriptional activity without affecting HIFα protein levels, creating a positive feedback loop. Furthermore, zebrafish deficiency in *med15* displayed decreased HIF activity and impaired tolerance to hypoxic stress. Functionally, MED15 deficiency attenuated the proliferation of colon and renal cancer cells *in vitro* and tumor growth *in vivo*. Mechanistically, MED15 acts upstream of carnitine palmitoyltransferase 1A (CPT1A), a key enzyme in fatty acid oxidation, ultimately promoting HIF-mediated LD accumulation. Disrupting the MED15-CPT1A axis impairs this process. These findings reveal a novel MED15-HIF-CPT1A axis that promotes LD formation, potentially contributing to hypoxic tumor progression.

Tumor hypoxia is a hallmark feature of solid tumors and is associated with aggressive tumor behavior, therapy resistance, and poor patient prognosis (1, 2, 3). Hypoxia-inducible factors (HIFs) are master regulators of cellular adaptation to low oxygen conditions (4, 5, 6, 7). HIFs promote the expression of genes involved in various processes, including angiogenesis, metabolic reprogramming, and cell survival, ultimately contributing to tumor progression (8, 9, 10, 11, 12, 13). One key aspect of metabolic reprogramming in hypoxic tumors is the altered lipid metabolism (14, 15, 16, 17, 18). While normal cells primarily rely on oxidative phosphorylation for energy production, hypoxic tumors often exhibit a shift towards aerobic glycolysis and increased lipid storage (16, 19). However, the precise mechanisms underlying lipid droplet (LD) accumulation in hypoxic tumors remain elusive.

Mediator complex, located in the nucleus, is evolutionarily conserved from yeast (with 25 subunits) to humans (with 30 subunits) (20, 21). In humans, the Mediator complex consists of head, middle, tail, and a dissociable kinase module (with 4 subunits), with a total size of 1.4 MDa (22). Acting as a bridge molecule between transcription factors and RNA polymerase Ⅱ, the Mediator complex plays multiple roles in regulating the activity of RNA polymerase Ⅱ by interacting with various transcription factors (23, 24, 25). Mediator complex subunit 15 (MED15), a component of the tail module and composed of 788 amino acids, has emerged as a potential regulator of lipid metabolism (26, 27, 28). A recent study demonstrated that MED15 cooperates with sterol regulatory element-binding protein 1 (SREBP1) to promote the expression of lipogenic genes, including those encoding stearoyl-CoA-desaturases (29). In *Caenorhabditis elegans*, MED15 deficiency leads to glucose-induced accelerated aging, while its presence promotes longevity under low temperatures, potentially through mechanisms involving enhanced lipidostasis (26, 27). However, our understanding of MED15 function in higher organisms remains incomplete. While some studies suggest an oncogenic role for MED15, potentially linking it to the development of breast, prostate, and renal cancers, the underlying mechanisms are poorly understood (30, 31, 32, 33).

This study investigates the role of MED15 in the regulation of LD accumulation under hypoxia. We demonstrate that MED15 acts as a key regulator of HIF signaling. Interestingly, hypoxia itself induces MED15 expression, creating a novel positive feedback loop that likely contributes to LD accumulation and potentially fuels the progression of hypoxic tumors. These findings shed light on a previously unknown mechanism underlying hypoxic tumor metabolism and may offer a new therapeutic target for the treatment of hypoxic cancers.

## Results

### MED15 depletion suppresses HIF transcriptional activity without affecting its protein levels

**Figure 1:**
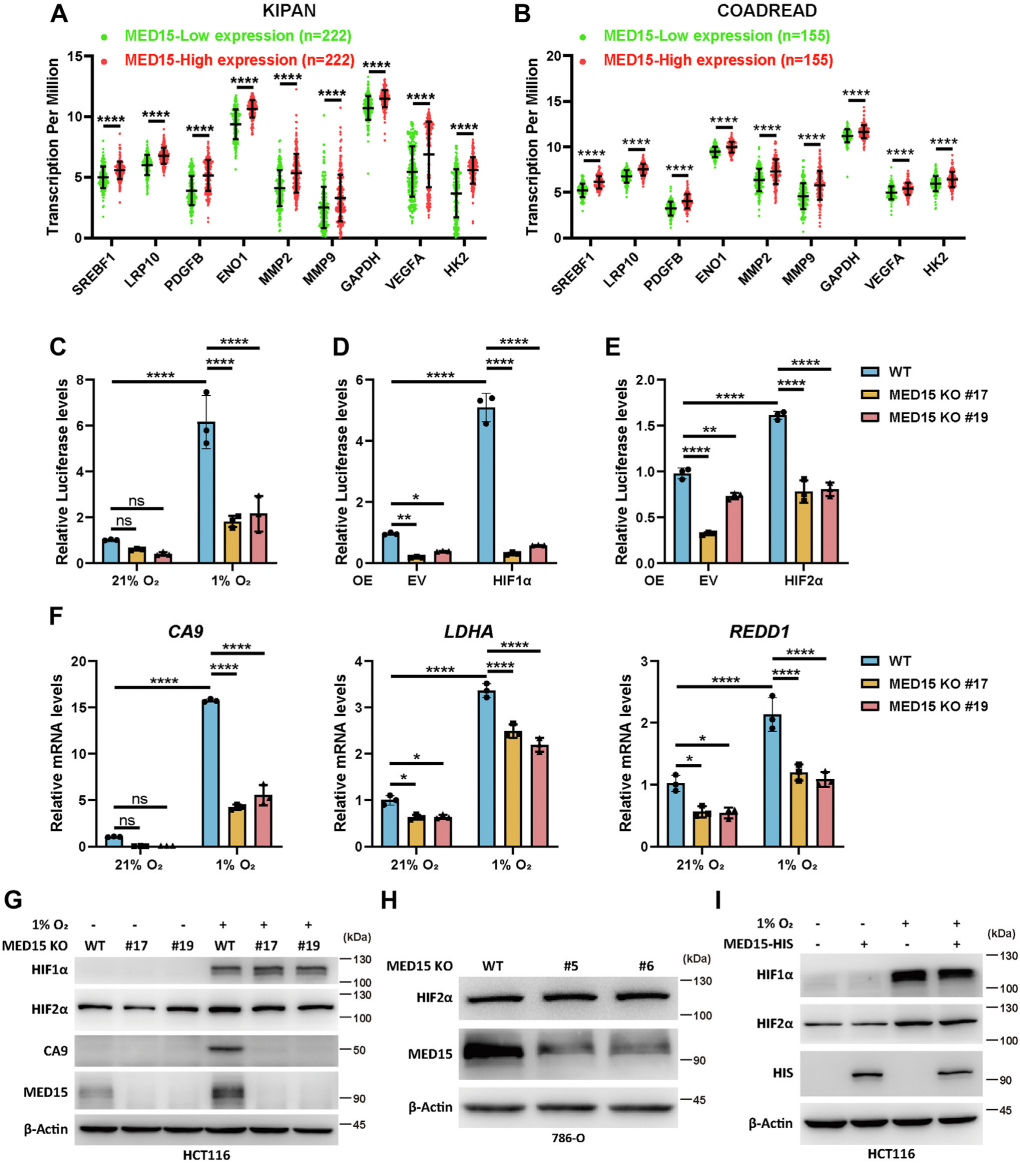
**MED15 positively regulates HIF transcriptional activity.***A* and *B*, expression levels of HIF target genes in clinical cancer samples from the TCGA cohorts in the MED15 low expression group (*bottom* 25%) and high expression group (*top* 25%) are shown. ∗∗∗∗*p* < 0.0001 by Student's *t* test. *C*–*E*, luciferase reporter assays in HCT116 cells exposed to 21% O_2_ or 1% O_2_ for 24 h (*C*) or transfected with empty vector (EV), HIF1α (*D*), or HIF2α (*E*). The Firefly luciferase activity was normalized to the Renilla luciferase activity. Data are represented as means ± SD. n = 3 biologically independent extracts. ∗*p* < 0.05, ∗∗*p* < 0.01, ∗∗∗∗*p* < 0.0001 by two-way ANOVA with Tukey's multiple comparisons test. *F*, RT-qPCR analysis of *CA9*, *LDHA*, and *REDD1* in WT or MED15 KO (KO #17, KO #19) HCT116 cells exposed to 21% O_2_ or 1% O_2_ for 24 h. Data are represented as means ± SD. n = 3 biologically independent extracts. ∗*p* < 0.05, ∗∗∗∗*p* < 0.0001 by two-way ANOVA with Tukey's multiple comparisons test. *G*, Western blot of HIF1α, HIF2α, and CA9 in HCT116 WT or MED15 KO cells exposed to 21% O_2_ or 1% O_2_ for 24 h. *H*, Western blot of HIF2α in 786-O WT or MED15 KO cells. *I*, Western blot of HIF1α and HIF2α in HCT116 cells transfected with EV or HIS-tagged MED15. Cells were exposed to 21% O_2_ or 1% O_2_ for 24 h. CA9, carbonic anhydrase 9; COADREAD, Colon adenocarcinoma/Rectum adenocarcinoma Esophageal carcinoma; HIF, hypoxia-inducible factor; KIPAN, Pan-kidney cohort; LDHA, lactate dehydrogenase A; MED15, Mediator complex subunit 15; ns, not significant; REDD1, protein regulated in development and DNA damage response 1.

**Supplemental Figure 1:**
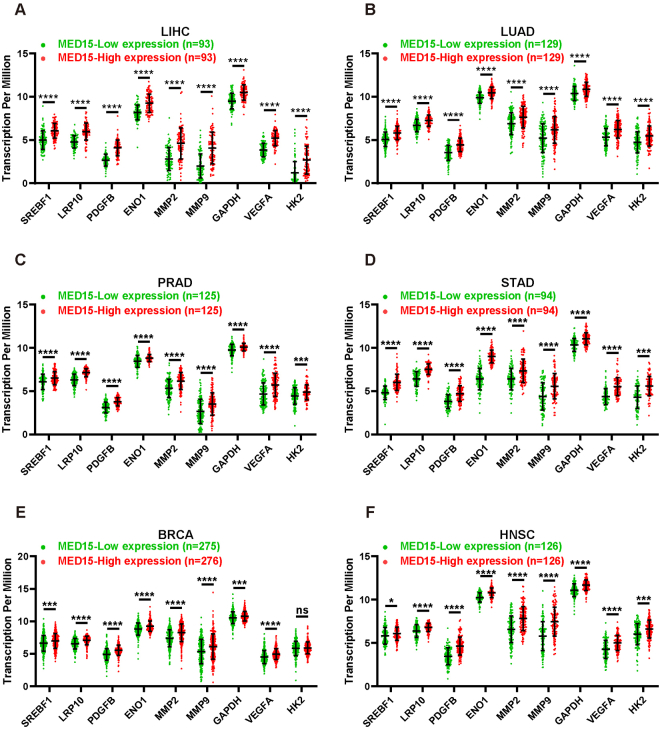


**Supplemental Figure 2:**
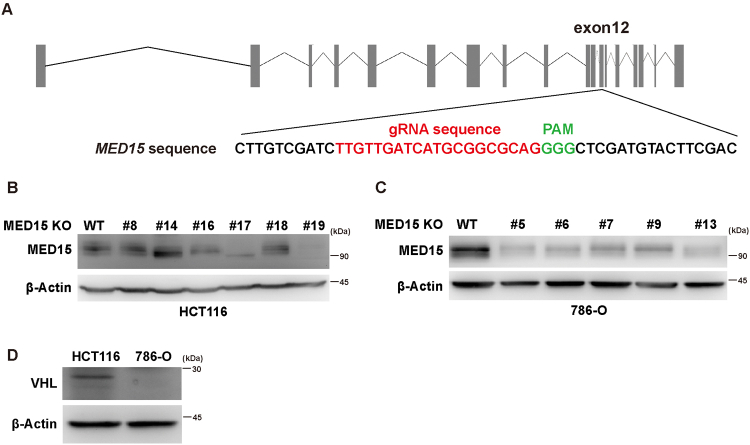


**Supplemental Figure 3:**
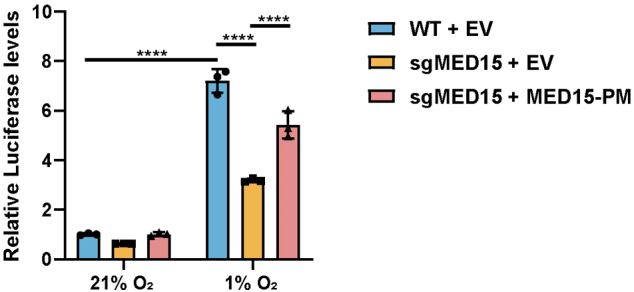


**Supplemental Figure 4:**
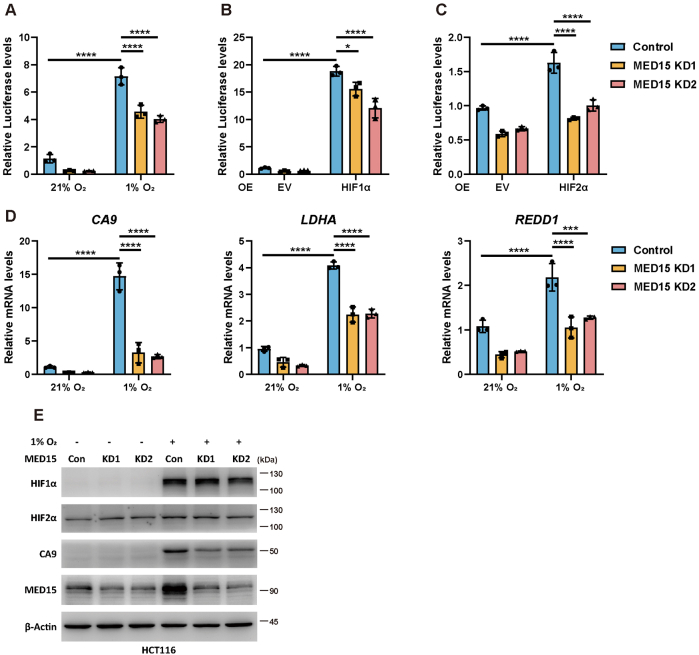


Analysis of The Cancer Genome Atlas (TCGA) datasets revealed a positive correlation between MED15 expression and the expression of HIF target genes, including *ENO1*, *HK2*, and *VEGFA*, in renal and colorectal cancers (Fig. 1, *A* and *B*). Similar positive correlations were observed in other solid cancers (Fig. S1, *A*–*F*), suggesting that MED15 may positively regulate HIF signaling. To investigate this further, we generated MED15 KO colon cancer HCT116 cells and renal cell carcinoma pVHL-mutant 786-O cells using CRISPR/Cas9 technology (Fig. S2, *A*–*D*). To assess HIF transcriptional activity, WT and MED15 KO HCT116 cells were cotransfected with a hypoxia response element (HRE)-driven HIF luciferase reporter (p2.1) and a control reporter (pSV40-Renilla) (7). Following transfection, cells were exposed to either 21% O_2_ or 1% O_2_ for 24 h. Notably, MED15 knockout significantly inhibited HIF transcriptional activity under hypoxic conditions (Fig. 1*C*). To demonstrate the specificity of the MED15-guide RNA (gRNA) site, we constructed a plasmid (MED15-PM) in which the gRNA site of MED15 was mutated while keeping the amino acid sequence unchanged. MED15-PM overexpression partially rescued the decrease in HIF activity caused by MED15 knockout, suggesting gRNA site specificity (Fig. S3). The role of MED15 in regulating HIF activity was further validated by the decreased transcriptional activity observed upon HIF1α or HIF2α overexpression in MED15 KO cells (Fig. 1, *D* and *E*). Similar results were obtained in HCT116 cells stably expressing shRNA targeting MED15 (Fig. S4, *A*–*C*).

Consistent with these findings, MED15 loss led to reduced mRNA levels of HIF-mediated genes, including *CA9*, *LDHA*, and *REDD1*, following hypoxia treatment (Figs. 1*F* and S4*D*). Western blot assay confirmed that CA9 expression, a well-characterized HIF target, was significantly downregulated under hypoxia upon MED15 deletion. Importantly, HIF1α and HIF2α protein levels remained unchanged (Figs. 1*G* and S4*E*). Furthermore, MED15 deficiency did not affect endogenous HIF2α protein levels in 786-O cells (Fig. 1*H*), and MED15 overexpression did not alter HIF1α or HIF2α protein levels (Fig. 1*I*). Collectively, these findings demonstrate that MED15 acts as a positive regulator of HIF signaling and transcriptional activity.

### *med15* deficiency impairs hypoxia response and tolerance in zebrafish

**Figure 2:**
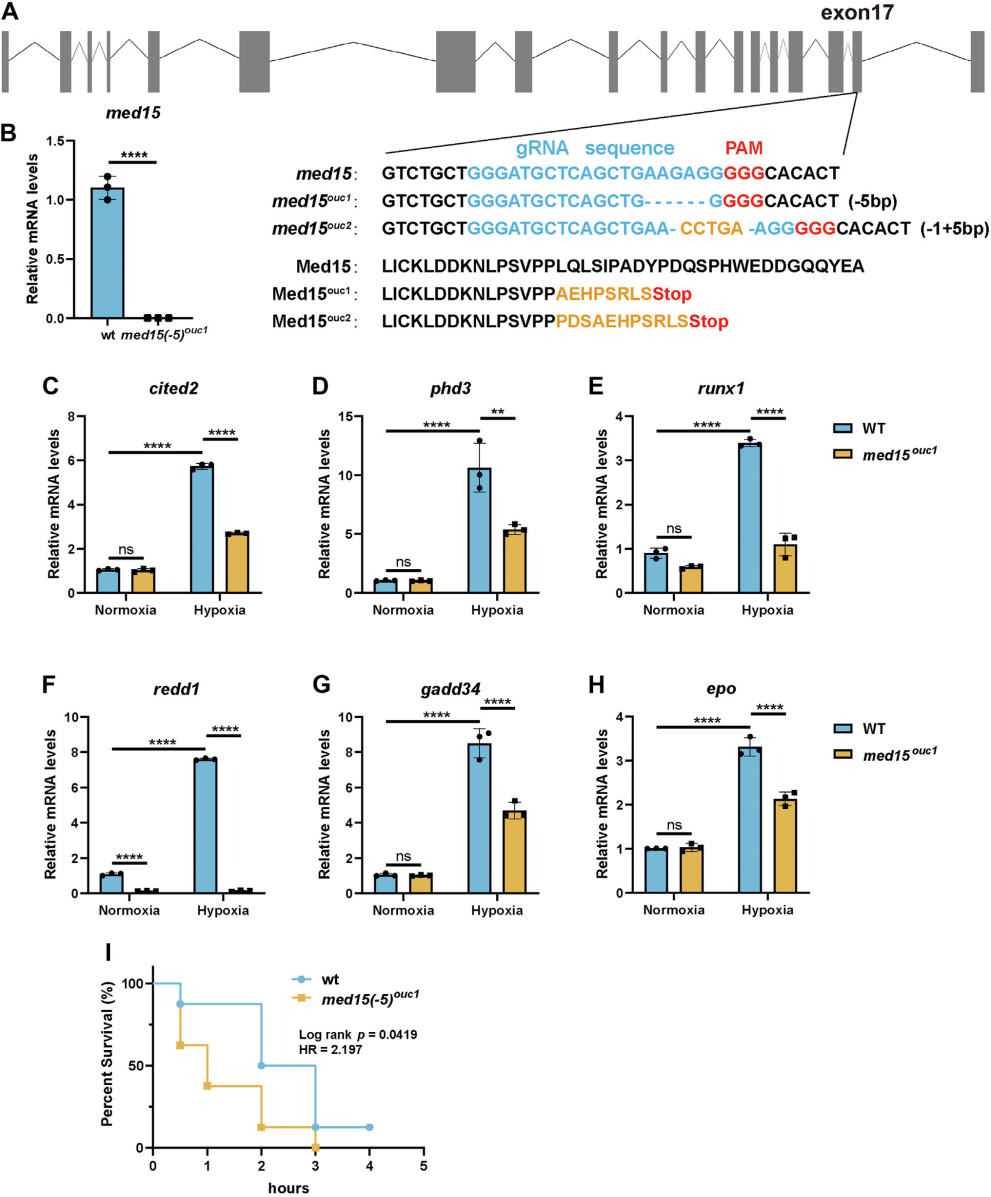
**Disruption of *med15* in zebrafish suppresses hypoxia signaling.***A*, information for constructing *med15*-deficient zebrafish using CRISPR/Cas9 system. *B*, RT-qPCR analysis of the mRNA levels of *med15* in WT or *med15*-deficient (*med15*^*ouc1*^) zebrafish larvae (3 dpf). Data are represented as means ± SD. n = 3 biologically independent extracts. ∗∗∗∗*p* < 0.0001 by Student's *t* test. *C*–*H*, RT-qPCR analysis of *cited2* (*C*), *phd3* (*D*), *runx1* (*E*), *redd1* (*F*), *gadd34* (*G*), and *epo* (*H*) mRNA levels in WT or *med15*-deficient (*med15*^*ouc1*^) zebrafish larvae (3 dpf) under normoxia (21% O_2_) or hypoxia (6% O_2_) for 10 h. Data are represented as means ± SD. n = 3 biologically independent extracts. ∗∗*p* < 0.01, ∗∗∗∗*p* < 0.0001 by two-way ANOVA with Tukey's multiple comparisons test. *I*, Kaplan-Meier survival analysis for WT (n = 8) and *med15*-null (n = 8) adult zebrafish under 5% hypoxia conditions by log rank test. *cited2*, CBP/p300-interacting transactivator 2; *epo*, erythropoietin; *gadd34*, growth arrest and DNA damage-inducible protein 34; HR, hazard ratio; ns, not significant; *med15*, Mediator complex subunit 15; *phd3*, prolyl hydroxylase; *redd1*, protein regulated in development and DNA damage response 1; *runx1*, runt-related transcription factor 1.

**Supplemental Figure 5:**
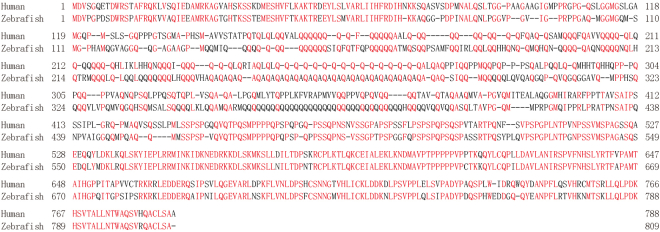


Zebrafish *med15* exhibits a high degree of evolutionary conservation with human MED15, sharing 70.65% sequence similarity (Fig. S5). To elucidate the role of MED15 in the hypoxia response *in vivo*, we utilized the CRISPR/Cas9 system to generate zebrafish with a null mutation in *med15*. DNA sequencing confirmed successful generation of two *med15* mutant alleles, *med15*^*ouc1*^ and *med15*^*ouc2*^ (Fig. 2*A*). Real-time quantitative PCR (RT-qPCR) analysis demonstrated a significant decrease in *med15* mRNA levels in the corresponding mutants (Fig. 2*B*).

Building on our *in vitro* observations, we investigated the effect of *med15* deficiency on the hypoxia response *in vivo*. Hypoxia treatment induced the expression of HIF target genes, including *cited2*, *phd3*, *runx1*, *redd1*, *gadd34*, and *epo*, in WT zebrafish embryos. However, this hypoxic induction was significantly blunted in *med15* mutant embryos (Fig. 2, *C*–*H*). Subsequently, we evaluated the hypoxic tolerance of adult mutant zebrafish using a previously established hypoxic aquarium system (34). Notably, *med15*-deficient zebrafish displayed significantly reduced survival under hypoxic conditions compared to wt controls (Fig. 2*I*). These data collectively suggest a critical role for *med15* in mediating the hypoxia response and promoting tolerance to hypoxic stress in zebrafish.

### *MED15* is a novel HIF target gene

**Figure 3:**
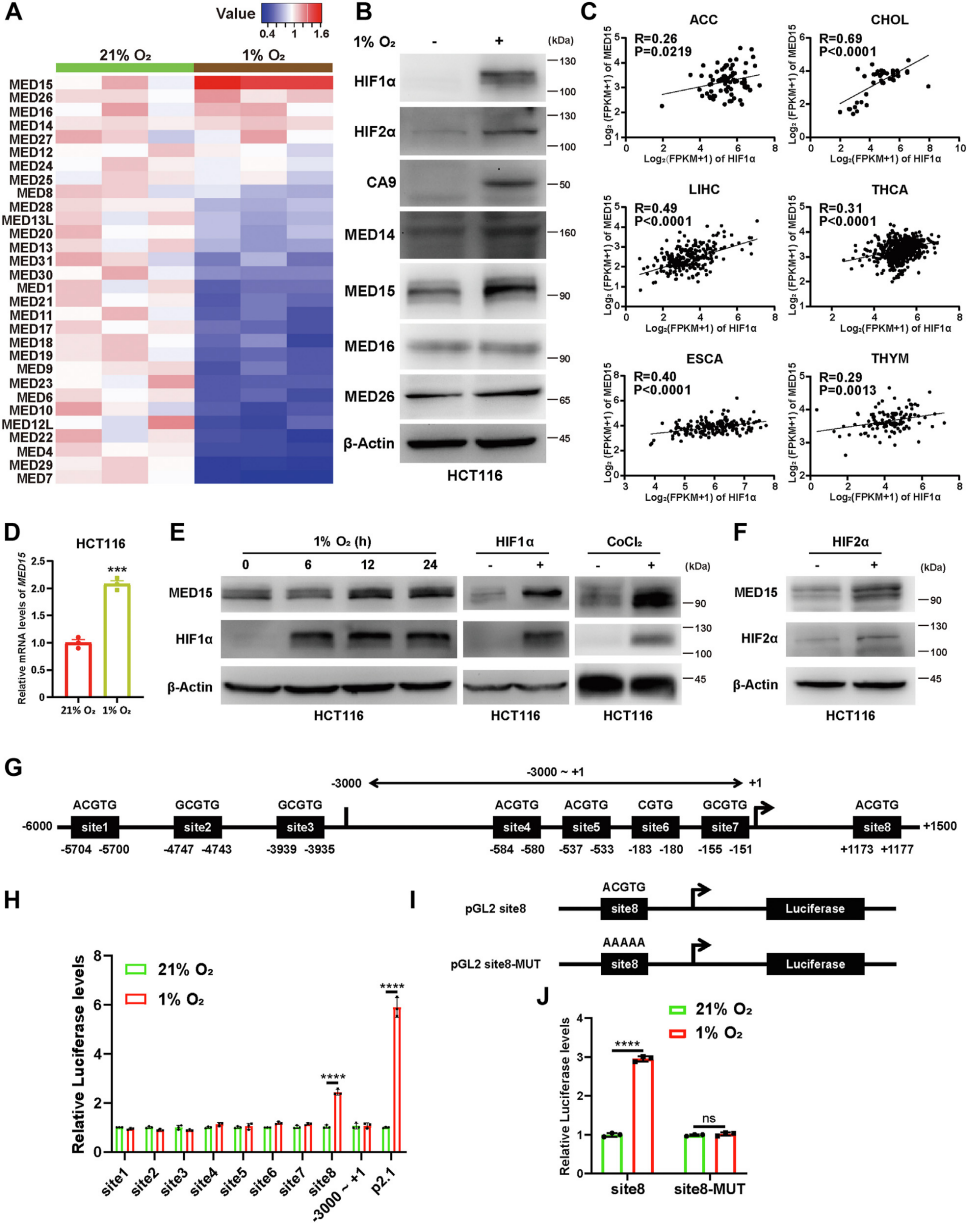
**MED15 is a HIF target gene.***A*, HCT116 cells were exposed to either 21% O_2_ or 1% O_2_ for 24 h. RNA sequencing was performed to analyze the transcript levels of Mediator complex subunits. *B*, HCT116 cells were exposed to 21% O_2_ or 1% O_2_ for 24 h, and Western blot assay was performed with indicated antibodies. *C*, correlations analysis of MED15 and HIF1α mRNA levels in cancer samples from TCGA is shown. The sample correlation coefficient (R) and corresponding *p* values are indicated. *D*, RT-qPCR analysis of *MED15* mRNA levels in HCT116 cells exposed to 21% O_2_ or 1% O_2_ for 24 h. Data are represented as means ± SD. n = 3 biologically independent extracts. ∗∗∗*p* < 0.001 by Student's *t* test. *E*, HCT116 cells were exposed to 1% O_2_ for the indicated times, or transfected with HIF1α expression vector, or treated with CoCl_2_. Western blot analysis was then performed to evaluate MED15 protein levels. *F*, Western blot of MED15 protein in HCT116 cells transfected with empty vector or HIF2α expression vector. *G*, diagram of the MED15 promoter region (6000 base pairs upstream to 1500 base pairs downstream of the transcriptional start site (+1), NCBI reference sequence NC_000022.11: 20501610-20509109) analyzed for potential HREs. *H*, luciferase assay in HCT116 cells cotransfected with pSV40-Renilla and p2.1, different pGL2 HREs, or pGL2 -3000 ∼ +1 region and then exposed to 21% O_2_ or 1% O_2_ for 24 h. The Firefly luciferase activity was normalized to the Renilla luciferase activity. Data are represented as means ± SD. n = 3 biologically independent extracts. ∗∗∗∗*p* < 0.0001 by Student's *t* test. *I*, diagram of pGL2 site8 and pGL2 site8-MUT. *J*, luciferase assay in HCT116 cells cotransfected with pSV40-Renilla and pGL2 site8 or pGL2 site8-MUT and then exposed to 21% O_2_ or 1% O_2_ for 24 h. The Firefly luciferase activity was normalized to the Renilla luciferase activity. Data are represented as means ± SD. n = 3 biologically independent extracts. ∗∗∗∗*p* < 0.0001 by Student's *t* test. HIF, hypoxia-inducible factor; MED15, Mediator complex subunit 15; MUT, mutant; ns, not significant.

We conducted RNA sequencing of HCT116 cells exposed to either 21% O_2_ or 1% O_2_ for 24 h and analyzed the mRNA levels of the Mediator complex subunits. Interestingly, while most subunits showed a general decrease in mRNA levels, the expression of *MED15* was upregulated (Fig. 3*A*). Furthermore, Western blot analysis in HCT116 cells found a significant upregulation of MED15 protein level after 24 h of 1% O_2_ treatment (Fig. 3*B*). This finding prompted us to investigate whether HIF plays a role in MED15 induction within tumor contexts. We analyzed data from TCGA and identified a positive correlation between *MED15* and *HIF1α* mRNA levels across various cancers (Fig. 3*C*).

To validate these observations, we performed RT-qPCR assays in HCT116 cells exposed to either 21% O_2_ or 1% O_2_ for 24 h. As expected, MED15 mRNA levels were significantly upregulated under hypoxic conditions (Fig. 3*D*). We also observed an increase in the protein levels of MED15 following hypoxia treatment with indicated time (Fig. 3*E*). Furthermore, overexpression of either HIF1α or HIF2α also led to increased MED15 protein levels. Similarly, treatment with cobalt chloride (CoCl_2_), a known HIF stabilizer that inhibits prolyl hydroxylase, also resulted in MED15 protein upregulation (Fig. 3, *E* and *F*).

Next, we investigated whether *MED15* is a HIF target gene by analyzing potential HREs in MED15 promoter region using JASPAR (https://jaspar.elixir.no/) database. We identified eight possible HRE sites (Fig. 3*G*). Using the pGL2 reporter plasmid, we generated nine reporter plasmids by separately inserting a 3000 base pairs region upstream of transcriptional start site (−3000 ∼ +1) or nucleotide sequences containing different HRE sites into upstream of firefly luciferase coding sequence. HCT116 cells were cotransfected with pSV40-Renilla and nine different HRE reporter plasmids and exposed to either 21% O_2_ or 1% O_2_ for 24 h. Notably, only pGL2 reporter plasmid with site8 inserted showed significant activation by 1% O_2_ (Fig. 3*H*). Furthermore, the mutant of site8 (5′-ACGTG-3′ to 5′-AAAAA-3′) completely lost hypoxia-induced luciferase activity (Fig. 3, *I* and *J*). Collectively, these findings strongly suggest that *MED15* is a HIF target gene.

### MED15 promotes the growth of colon and ccRCC tumors

**Supplemental Figure 6:**
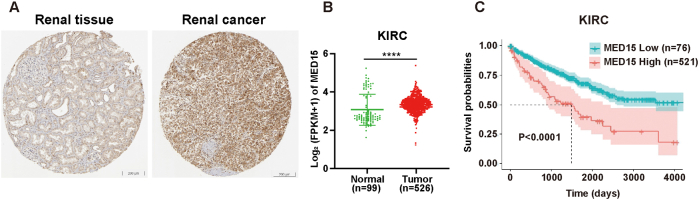


Analysis of immunohistochemical (IHC) data from The Human Protein Atlas (https://www.proteinatlas.org/) database (Fig. S6*A*) and mRNA levels from TCGA (Fig. S6*B*) revealed a high expression of MED15 in renal cancer tissues compared to normal tissues. Meanwhile, Kaplan–Meier analysis of the TCGA dataset demonstrated a significant correlation between elevated MED15 mRNA levels and poor overall survival in patients with kidney renal clear cell carcinoma (KIRC) (Fig. S6*C*).

**Figure 4:**
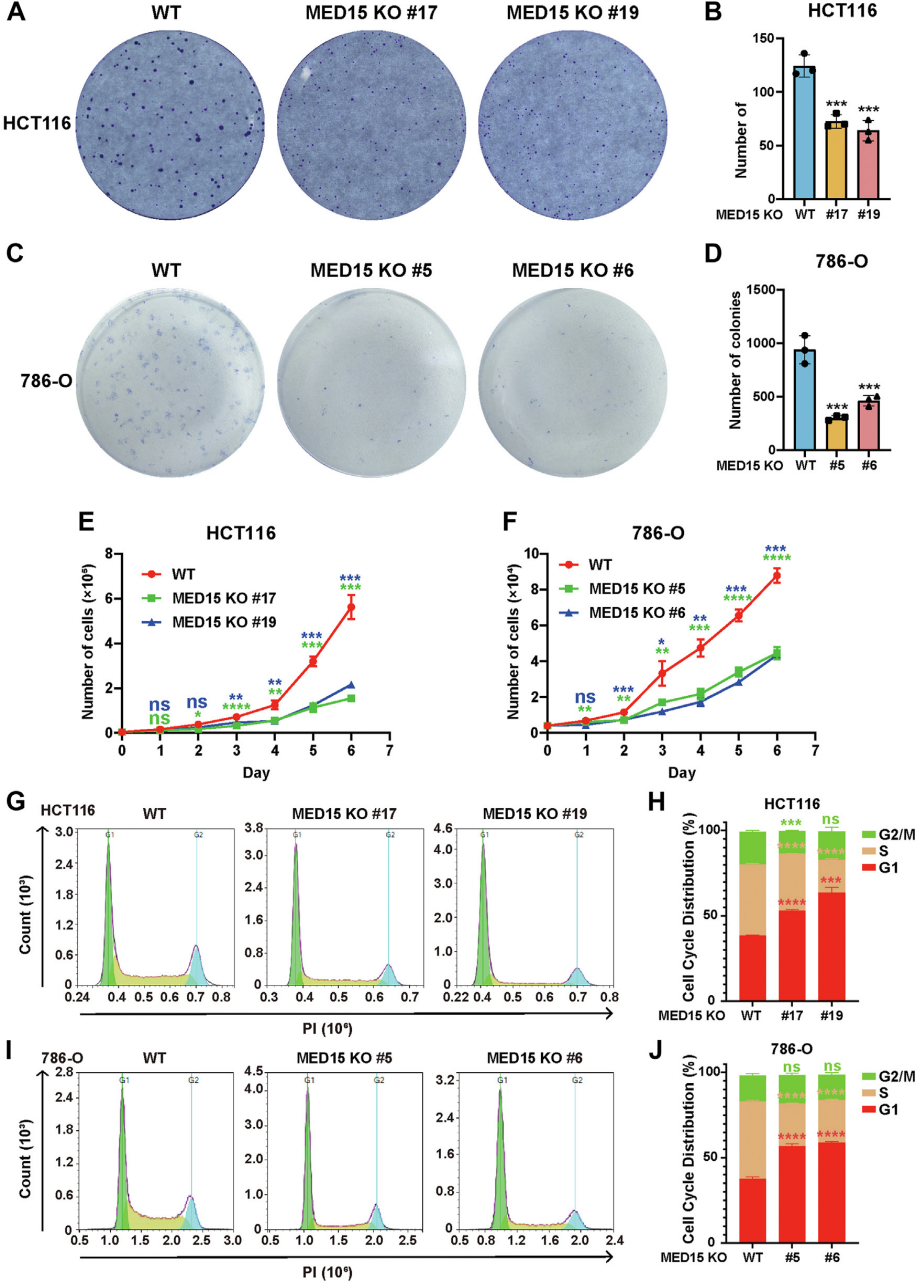
**MED15 depletion suppresses the proliferation of tumor cells *in vitro*.***A*–*D*, colony formation assay of WT and MED15 KO HCT116 cells (KO #17, KO #19) and 786-O cells (KO #5, KO #6). Cells were cultured for 9 days. Representative images from three independent experiments are shown in (*A* and *C*). Quantification of colony numbers is shown in (*B* and *D*). Data are represented as means ± SD. n = 3 biologically independent repeats. ∗∗∗*p* < 0.001 by one-way ANOVA with Sidak's *t* test. *E* and *F*, cell proliferation curves of WT and MED15 KO HCT116 cells (KO #17, KO #19) and 786-O cells (KO #5, KO #6). Cell numbers were determined by cell counter. Data are represented as means ± SD. n = 3 biologically independent extracts. ∗*p* < 0.05, ∗∗*p* < 0.01, ∗∗∗*p* < 0.001, ∗∗∗∗*p* < 0.0001 by one-way ANOVA with Sidak's *t* test. *G*–*J*, flow cytometry analysis of cell cycle distribution in WT and MED15 KO HCT116 cells (KO #17, KO #19) and 786-O cells (KO #5, KO #6). Representative images from three independent experiments are shown in (*G* and *I*). Statistics of cell cycle distribution are shown in (*H* and *J*). Data are represented as means ± SD. n = 3 biologically independent extracts. ∗∗∗*p* < 0.001, ∗∗∗∗*p* < 0.0001 by one-way ANOVA with Sidak's *t* test. ns, not significant; MED15, Mediator complex subunit 15.

**Figure 5:**
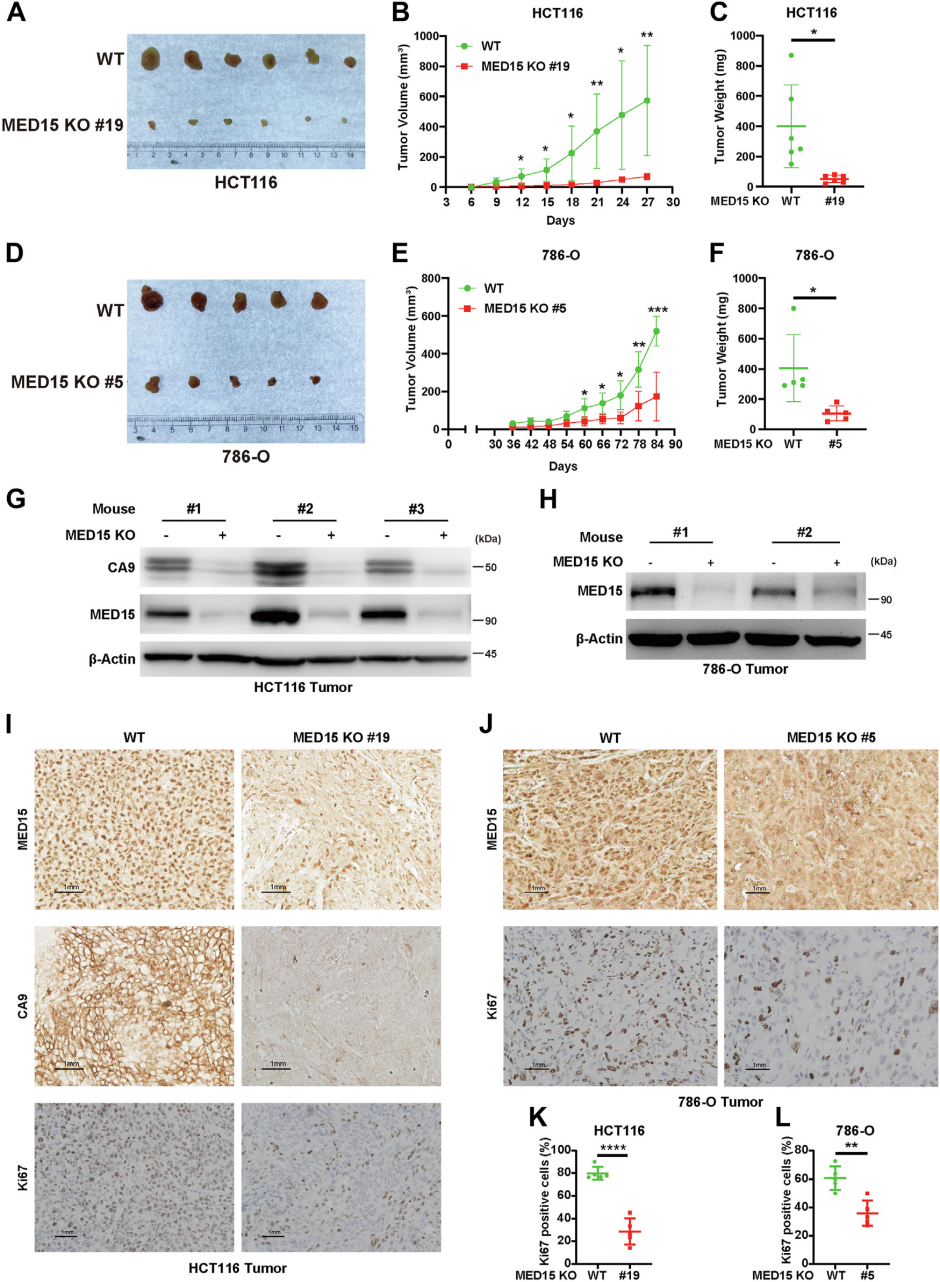
**Knockout of MED15 suppresses tumor growth *in vivo*.***A*–*C*, WT and MED15 KO (KO #19) HCT116 cells were transplanted into the armpits of nude mice, and tumor volume was measured every 3 days. Tumor image (*A*), growth curves (*B*), and weight (*C*) are shown (n = 6). Data are represented as means ± SD. ∗*p* < 0.05, ∗∗*p* < 0.01 by Student's *t* test. *D*–*F*, WT and MED15 KO (KO #5) 786-O cells were transplanted into the armpits of nude mice, and tumor volume was measured every six days starting from the 36th day. Tumor image (*D*), growth curves (*E*), and weight (*F*) are shown (n = 5). Data are represented as means ± SD. ∗*p* < 0.05, ∗∗*p* < 0.01, ∗∗∗*p* < 0.001 by Student's *t* test. *G*, protein levels of MED15 and CA9 were determined by Western blot using the lysates from six independent tumors in (*A*). *H*, protein levels of MED15 were determined by Western blot using the lysates from four independent tumors of (*D*). *I*, representative IHC images of MED15, CA9, and Ki67 using the paraffin section of tumors in (*A*) are shown. *J*, representative IHC images of MED15 and Ki67 using the paraffin section of tumors in (*D*) are shown. *K* and *L*, Ki67-positive cells were counted from the images in (*I*) (n = 5) and (J) (n = 5). Data are represented as means ± SD. ∗∗*p* < 0.01, ∗∗∗∗*p* < 0.0001 by Student's *t* test. CA9, carbonic anhydrase 9; Ki67, proliferation marker protein Ki-67; MED15, Mediator complex subunit 15.

MED15 deficiency in colorectal (HCT116) and renal (786-O) cancer cell lines resulted in a significant decrease in colony formation (Fig. 4, *A*–*D*). Additionally, cell growth curve analysis confirmed that loss of MED15 significantly inhibited tumor cell proliferation *in vitro* (Fig. 4, *E* and *F*). Flow cytometry analysis of cell cycle distribution revealed G1 phase arrest upon MED15 knockout (Fig. 4, *G*–*J*). Collectively, these data indicate that MED15 plays a critical role in promoting cancer cell proliferation *in vitro*. To assess the role of MED15 in tumorigenesis *in vivo*, nude mice xenograft models were established using HCT116 and 786-O cells. Mice harboring MED15-KO tumors exhibited significantly suppressed tumor growth compared to the WT controls (Fig. 5, *A*–*F*). Western blot analysis confirmed efficient MED15 deletion in both HCT116 and 786-O xenograft tumors (Fig. 5, *G* and *H*). We observed a dramatic reduction in the protein levels of CA9, consistent with our *in vitro* findings. IHC analysis further supported the reduction in CA9 protein levels (Fig. 5*I*). Additionally, IHC staining revealed a negative impact of MED15 knockout on the expression of Ki67, a marker of cell proliferation (Fig. 5, *I*–*L*). These findings strongly support the notion that MED15 promotes tumor growth *in vivo*.

### Loss of MED15 impairs HIF-mediated lipid droplet accumulation

**Figure 6:**
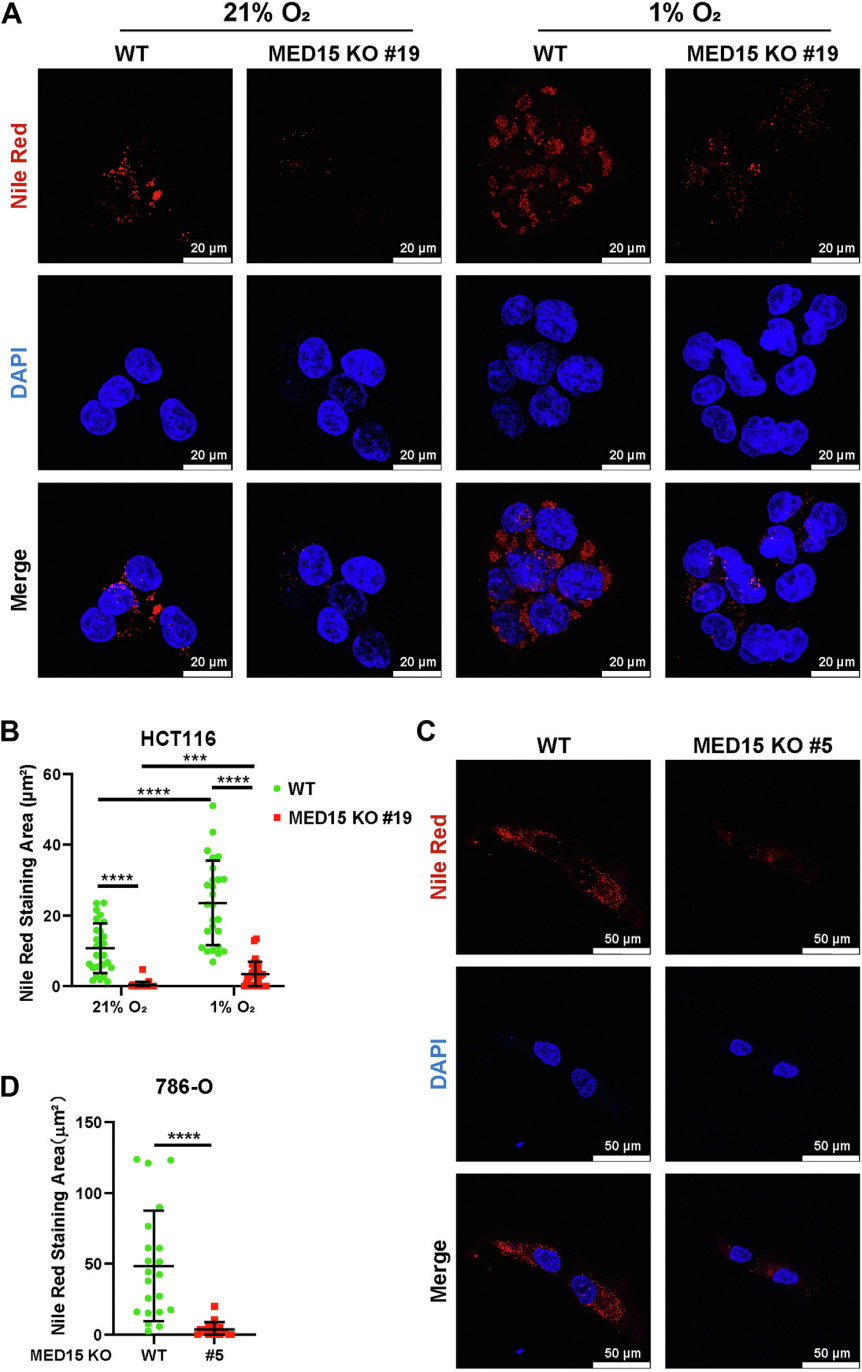
**Loss of MED15 suppresses HIF-induced lipid droplet accumulation.***A* and *B*, Nile Red and DAPI staining assay of WT and MED15 KO (KO #19) HCT116 cells under normoxia (21% O_2_) or hypoxia (1% O_2_) for 72 h. Representative images from three independent experiments are shown in (*A*). Quantification of Nile Red staining area is shown in (*B*). Data are represented as means ± SD. n = 27 (21% O_2_ WT); 25 (21% O_2_ MED15 KO #19); 26 (1% O_2_ WT); 30 (1% O_2_ MED15 KO #19). ∗∗∗*p* < 0.01, ∗∗∗∗*p* < 0.0001 by two-way ANOVA with Tukey's multiple comparisons test. *C* and *D*, Nile Red and DAPI staining assay of WT and MED15 KO (KO #5) 786-O cells. Representative images from three independent experiments are shown in (*C*). Quantification of Nile Red staining area is shown in (*D*). Data are represented as means ± SD. n = 21 (WT); 20 (MED15 KO #5). ∗∗∗∗*p* < 0.0001 by Student's *t* test. HIF, hypoxia-inducible factor; *med15*, Mediator complex subunit 15.

Since MED15 is required for lipid homeostasis in *C. elegans* (26, 27), we investigated its potential role in HIF-induced LD accumulation. Nile Red staining revealed a significant reduction in LD content in MED15 KO HCT116 cells compared to WT cells, under both normoxic and hypoxic conditions for 72 h (Fig. 6, *A* and *B*). Notably, while hypoxia induced LD accumulation in WT cells, MED15 knockout significantly blocked this response (Fig. 6*B*), suggesting MED15 is critical for hypoxia-induced LD formation. To further elucidate the role of MED15 in HIF-mediated LD regulation, we employed the pVHL-mutant 786-O cell line with constitutively high HIF2α levels due to pVHL mutations, which promote LD accumulation and contribute to ccRCC (35, 36, 37). We found that LD content was also markedly decreased in MED15-deficient 786-O cells (Fig. 6, *C* and *D*). These data indicate that MED15 plays an important role in HIF-mediated LD accumulation.

### Mutation of *med15* disrupts zebrafish lipid homeostasis

**Supplemental Figure 7:**
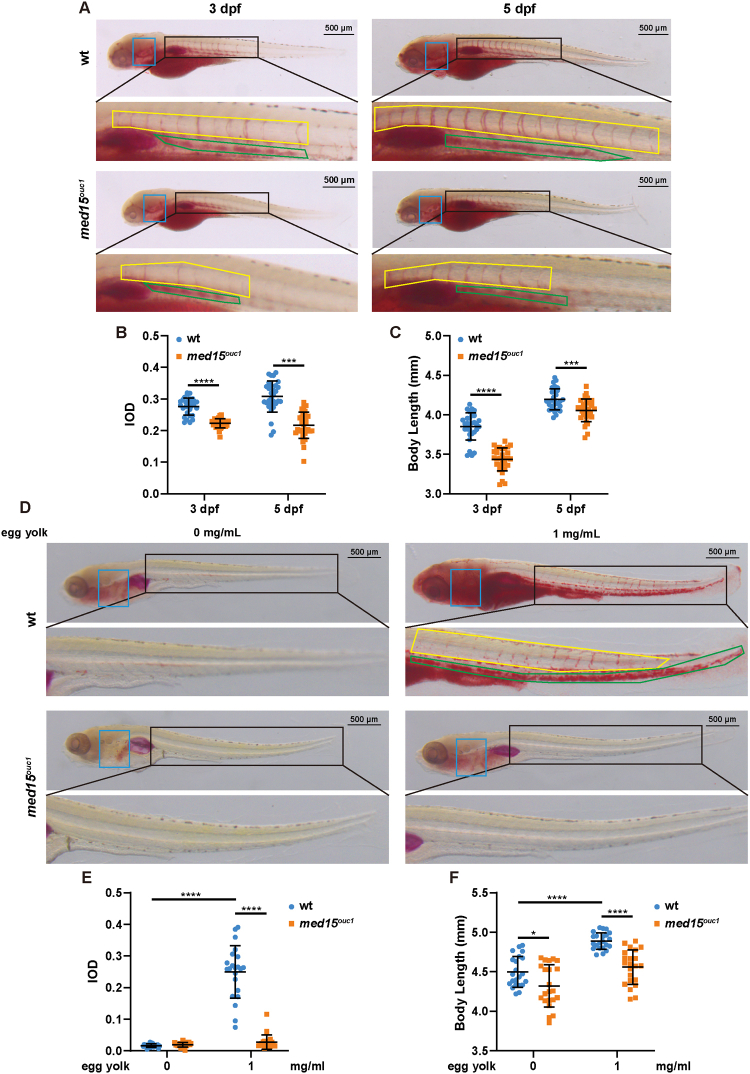


We further investigated the effect of *med15* on lipid homeostasis in zebrafish by Oil Red O staining assays. *med15*-deficient zebrafish displayed reduced lipid deposition in the head, internodal blood vessels, arteries, and arteries/veins at both 3 and 5 days postfertilization compared to wt zebrafish (Fig. S7, *A* and *B*). Notably, *med15* deficiency also resulted in shorter body length (Fig. S7*C*). Next, we established a hyperlipidemia model by feeding larvae with 1 mg/ml egg yolk for 5 days. *med15*-deficient larvae again displayed significantly reduced lipid accumulation in the head, blood vessels, and arteries/veins as assessed by Oil Red O staining intensity (IOD) (Fig. S7, *D* and *E*). Consistent with the above observation, these larvae displayed shorter body lengths than wt zebrafish (Fig. S7*F*). These findings collectively demonstrate that *med15* mutation disrupts lipid metabolism in zebrafish larvae. Additionally, *med15* deficiency appears to hinder zebrafish development, as evidenced by the reduced body length.

### Knocking out MED15 impairs lipid droplet accumulation by upregulating CPT1A expression

**Figure 7:**
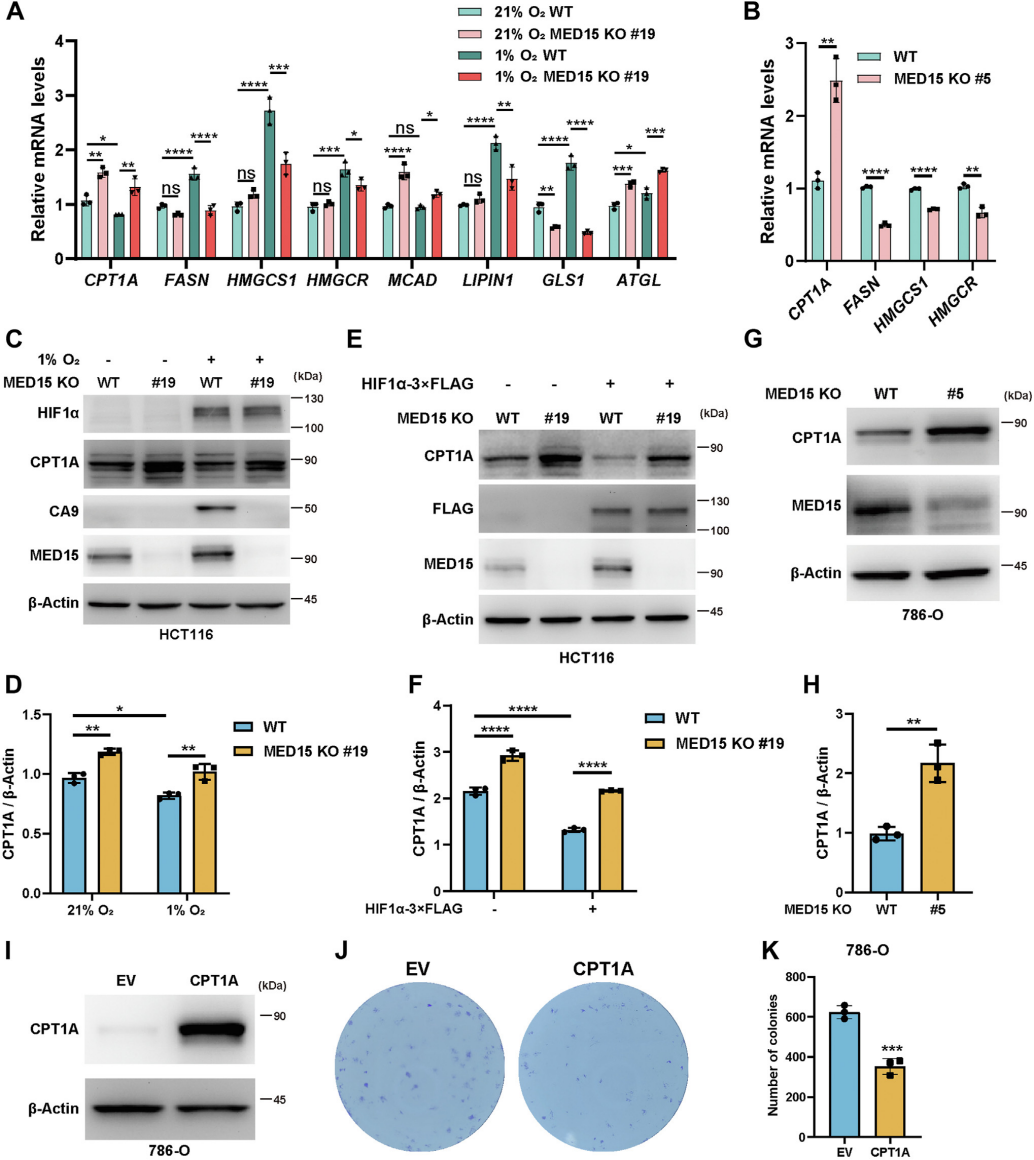
**Knocking out MED15 activates the expression of CPT1A.***A*, RT-qPCR analysis of the mRNA levels of *CPT1A*, *FASN*, *HMGCS1*, *HMGCR*, *MCAD*, *LIPIN1*, *GLS1*, and *ATGL* in WT or MED15 KO (KO #19) HCT116 cells exposed to 21% O_2_ or 1% O_2_ for 72 h. Data are represented as means ± SD. n = 3 biologically independent extracts. ∗*p* < 0.05, ∗∗*p* < 0.01, ∗∗∗*p* < 0.0001, ∗∗∗∗*p* < 0.0001 by two-way ANOVA with Tukey's multiple comparisons test. *B*, RT-qPCR analysis of the the mRNA levels of *CPT1A*, *FASN*, *HMGCS1*, and *HMGCR* in WT or MED15 KO (KO #5) 786-O cells. Data are represented as means ± SD. n = 3 biologically independent extracts. ∗∗*p* < 0.01, ∗∗∗∗*p* < 0.0001 by Student's *t* test. *C* and *D*, Western blot of indicated proteins in WT or MED15 KO (KO #19) HCT116 cells exposed to 21% O_2_ or 1% O_2_ for 72 h (*C*). Relative intensities of CPT1A were quantified (*D*). Data are represented as means ± SD. n = 3 biologically independent extracts. ∗*p* < 0.05, ∗∗*p* < 0.01 by two-way ANOVA with Tukey's multiple comparisons test. *E* and *F*, Western blot of indicated proteins in WT or MED15 KO (KO #19) HCT116 cells transfected with EV (−) or HIF1α-3 × FLAG (+) (*E*). Relative intensities of CPT1A were quantified (*F*). Data are represented as means ± SD. n = 3 biologically independent extracts. ∗∗∗∗*p* < 0.0001 by two-way ANOVA with Tukey's multiple comparisons test. *G* and *H*, Western blot of indicated proteins in WT or MED15 KO (KO #5) 786-O cells (*G*). Relative intensities of CPT1A were quantified (*H*). Data are represented as means ± SD. n = 3 biologically independent extracts. ∗∗*p* < 0.01 by Student's *t* test. *I*, Western blot assay of CPT1A confirmed that CPT1A protein level significantly upregulated after stable overexpression of CPT1A. *J* and *K*, colony formation assay of 786-O with stable overexpression of EV or CPT1A. Cells were cultured for 9 days. Representative images from three independent experiments are shown in (*J*). Quantification of colony numbers is shown in (*K*). Data are represented as means ± SD. n = 3 biologically independent repeats. ∗∗∗*p* < 0.001 by Student's *t* test. *ATGL*, adipose triglyceride lipase; *CPT1A*, carnitine palmitoyltransferase 1A; *FASN*, fatty acid synthase; EV, empty vector; *GLS1*, glutaminase 1; HIF, hypoxia-inducible factor; *HMGCS1*, 3-hydroxy-3-methylglutaryl-CoA synthase 1; *HMGCR*, 3hydroxy-3methylglutaryl-coenzyme A reductase; *MCAD*, medium-chain specific acyl-CoA dehydrogenase; MED15, Mediator complex subunit 15; ns, not significant.

**Supplemental Figure 8:**
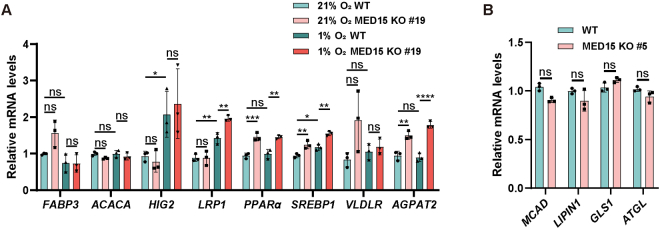


HIF promotes LD accumulation by enhancing fatty acid synthesis, increasing lipid uptake, inhibiting lipid breakdown, and reducing fatty acid oxidation (16, 18, 38, 39, 40). To elucidate the mechanism by which MED15 regulates HIF-mediated LD accumulation, we investigated its impact on lipid metabolism pathways. RT-qPCR analysis revealed that MED15 deficiency led to the downregulation of lipogenic genes such as *FASN*, *HMGCS1*, *HMGCR*, *LIPIN1*, and *GLS1*, especially under hypoxia conditions. Interestingly, loss of MED15 also induced the upregulation of genes involved in fatty acid β-oxidation and lipid hydrolysis, including *CPT1A*, *MCAD*, and *ATGL* (Figs. 7*A* and S8*A*). In 786-O cells, MED15 KO also led to increased *CPT1A* expression and decreased *FASN* expression (Figs. 7*B* and S8*B*). These findings suggest that MED15 may promote lipid accumulation by suppressing fatty acids β-oxidation or promoting fatty acid synthesis.

**Figure 8:**
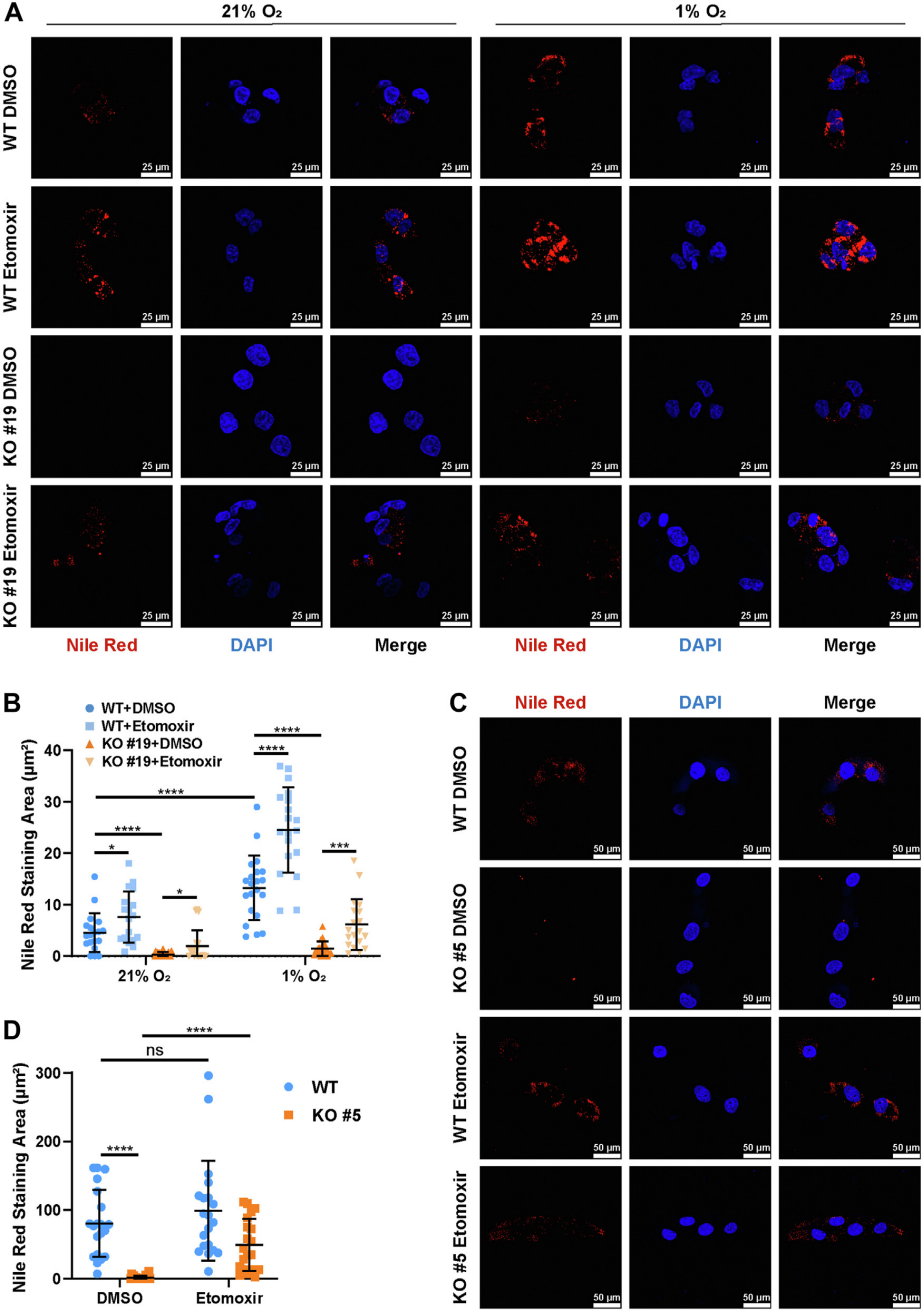
**Inhibiting CPT1A can rescue MED15 deficiency–dependent lipid droplet reduction.***A* and *B*, WT and MED15 KO HCT116 cells were exposed to 21% O_2_ or 1% O_2_ for 72 h in the presence of DMSO or Etomoxir (2.5 μM). LD and nuclei were staining with Nile Red and DAPI. Representative images from three independent experiments are shown in (*A*). Quantification of Nile Red staining area is shown in (*B*). Data are represented as means ± SD. n = 19 (21% O_2_ WT DMSO); 18 (21% O_2_ WT Etomoxir); 20 (21% O_2_ MED15 KO #19 DMSO); 20 (21% O_2_ MED15 KO #19 Etomoxir); 21 (1% O_2_ WT DMSO); 19 (1% O_2_ WT Etomoxir); 21 (1% O_2_ MED15 KO #19 DMSO); 19 (1% O_2_ MED15 KO #19 Etomoxir). ∗*p* < 0.05, ∗∗∗*p* < 0.001, ∗∗∗∗*p* < 0.0001 by two-way ANOVA with Tukey's multiple comparisons test. *C* and *D*, Nile Red and DAPI staining of WT and MED15 knockout 786-O treated with DMSO or Etomoxir (2.5 μM) for 72 h. Representative images from three independent experiments are shown in (*C*). Quantification of Nile Red staining area is shown in (*D*). Data are represented as means ± SD. n = 20 (WT DMSO); 20 (MED15 KO #5 DMSO); 20 (WT Etomoxir); 20 (MED15 KO #5 Etomoxir); ∗∗∗∗*p* < 0.0001 by two-way ANOVA with Tukey's multiple comparisons test. MED15, Mediator complex subunit 15; ns, not significant.

**Supplemental Figure 9:**
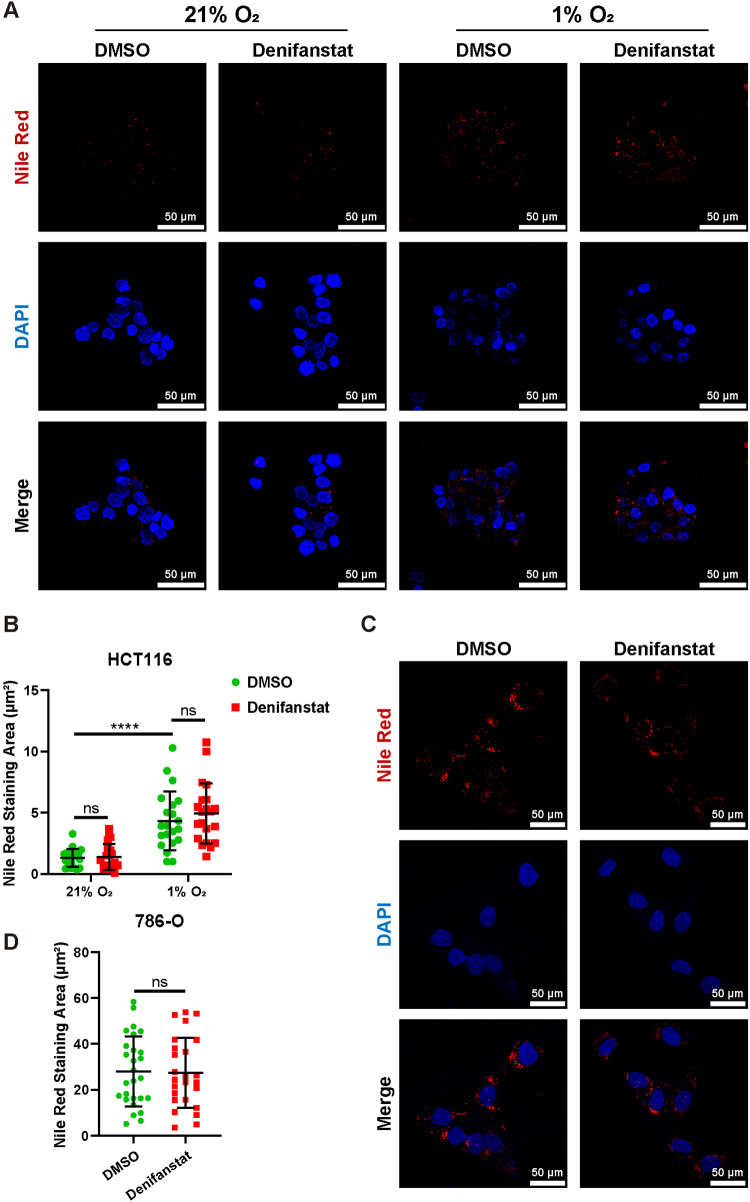


**Supplemental Figure 10:**
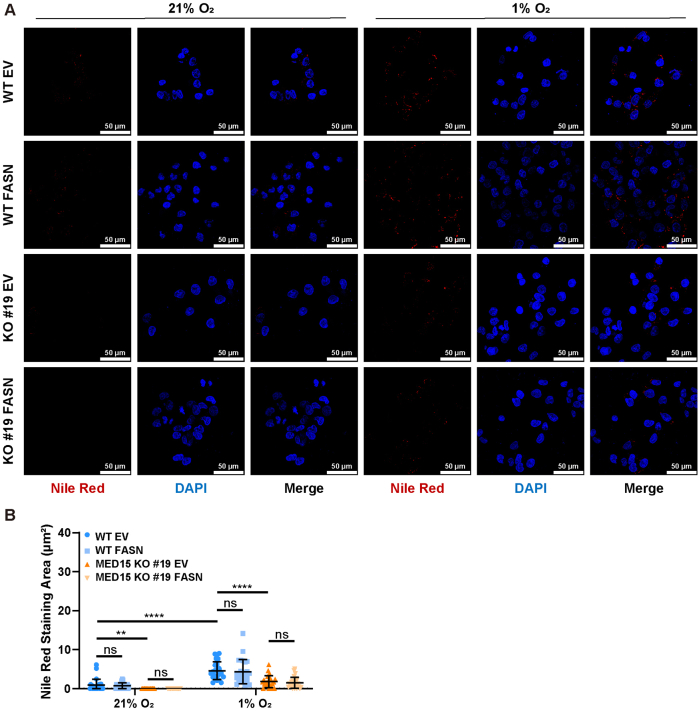


**Supplemental Figure 11:**
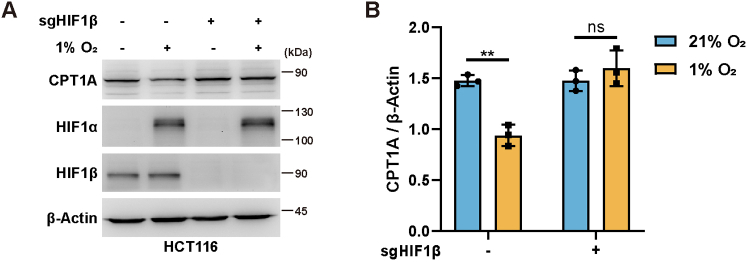


**Supplemental Figure 12:**
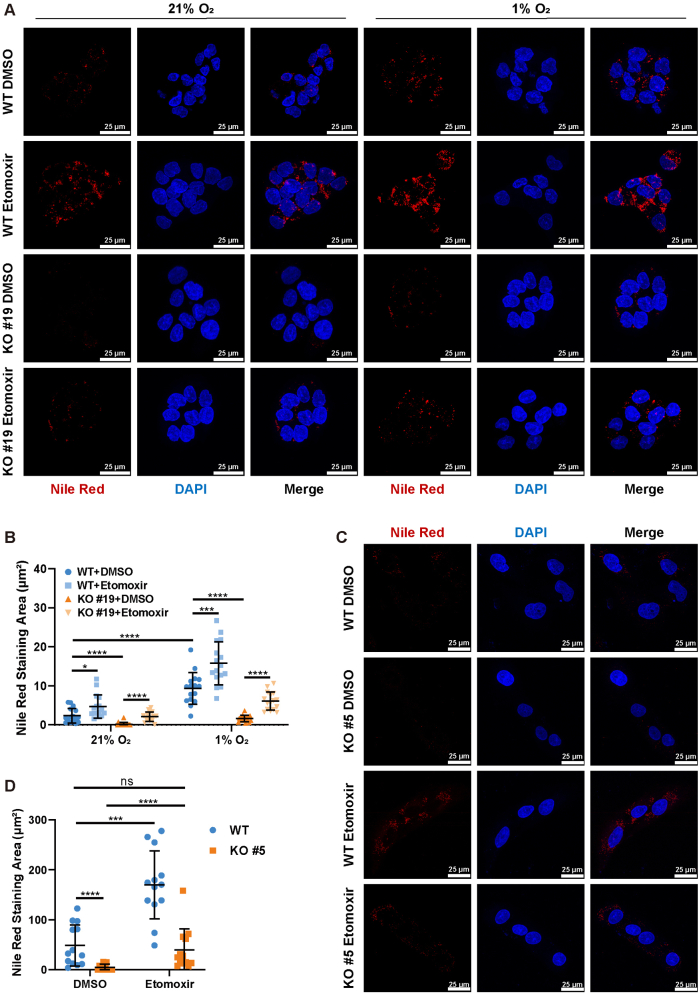


Previous studies have implicated MED15 in SREBP1-dependent lipogenesis gene expression. Depletion of MED15 downregulates SREBP1a-dependent FASN expression in human cells. Consequently, we hypothesized that the impairment of hypoxia-induced lipid accumulation upon MED15 deficiency might be mediated by alterations in fatty acid synthesis. However, inhibition of FASN with Denifanstat failed to mitigate the accumulation of LD under hypoxic conditions (Fig. S9, *A* and *B*). Additionally, 786-O cells, which also exhibit VHL deficiency, showed no significant decrease in LD content upon Denifanstat treatment (Fig. S9, *C* and *D*). Furthermore, overexpression of FASN in MED15 KO HCT116 cells did not rescue the reduction in LD (Fig. S10, *A* and *B*). These results suggest that alterations in fatty acid synthesis are unlikely to be the primary mechanism by which MED15 contributes to hypoxia-induced LD accumulation. Notably, CPT1A protein levels were elevated in both HCT116 and 786-O cells upon MED15 deficiency (Fig. 7, *C*–*H*). Furthermore, overexpression of CPT1A inhibits 786-O cells proliferation *in vitro* (Fig. 7, *I*–*K*). As the rate-limiting enzyme for mitochondrial fatty acid transport, the expression of CPT1A is diminished by HIFs, which contributes to hypoxia-mediated LD accumulation (8). Consistently, our study revealed that HIF1β knockout abolished the inhibition of hypoxia on CPT1A protein levels, further confirming that hypoxia impairs CPT1A expression by activating HIFs (Fig. S11, *A* and *B*). We hypothesized that MED15 deficiency disrupts lipid homeostasis by enhancing CPT1A expression. To test this hypothesis, we cultured WT and MED15 KO HCT116 cells under normoxia (21% O_2_) or hypoxia (1% O_2_) for 72 h with either DMSO or Etomoxir, a known CPT1A inhibitor (41, 42). As expected, MED15 knockout significantly attenuated hypoxia-induced LD accumulation, an effect reversed by both 2.5 μM and 40 μM Etomoxir treatment (Figs. 8, *A* and *B* and S12, *A* and *B*). Similar results were observed in 786-O cells (Figs. 8, *C* and *D* and S12, *C* and *D*).

**Supplemental Figure 13:**
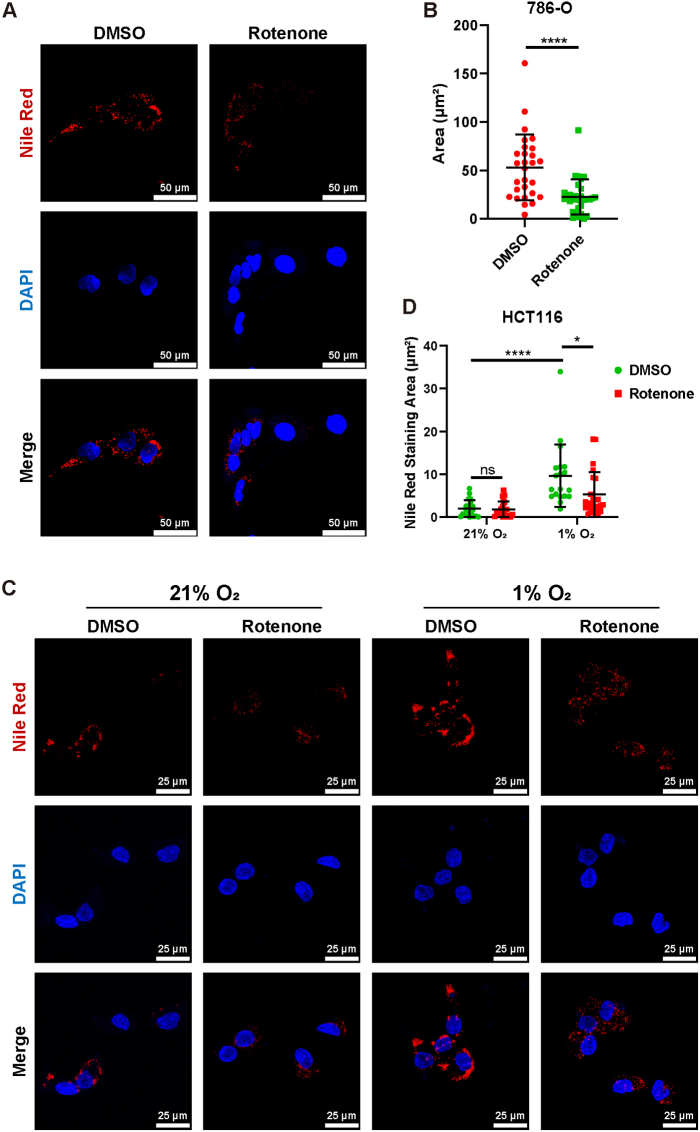


However, several studies have reported that Etomoxir, at concentrations exceeding 5 μM, inhibits both adenine nucleotide translocase (ANT) and NADH: ubiquinone oxidoreductase (complex I) (43, 44). To investigate whether these off-target effects contribute to Etomoxir's observed effects on LD homeostasis, we examined the impact of directly inhibiting ANT and complex I. 786-O cells were treated with DMSO, the ANT inhibitor agaric acid, or the complex I inhibitor Rotenone for 72 h (45, 46, 47). Nile Red staining assay revealed no significant increase in LD content in cells treated with either inhibitor (Figs. S13, *A* and *B* and S14, *A* and *B*). Similar results were observed in HCT116 cells (Fig. S13, *C* and *D*). These findings indicate that inhibiting ANT or complex I alone does not significantly alter LD accumulation, suggesting that these off-target effects are unlikely to be the primary mechanism underlying Etomoxir's impact on LD homeostasis.

Altogether, these data indicate that MED15 participates in maintaining lipid homeostasis through the HIF–CPT1A axis.

### MED15 enhances HIF1 binding to the HREs of target genes

**Figure 9:**
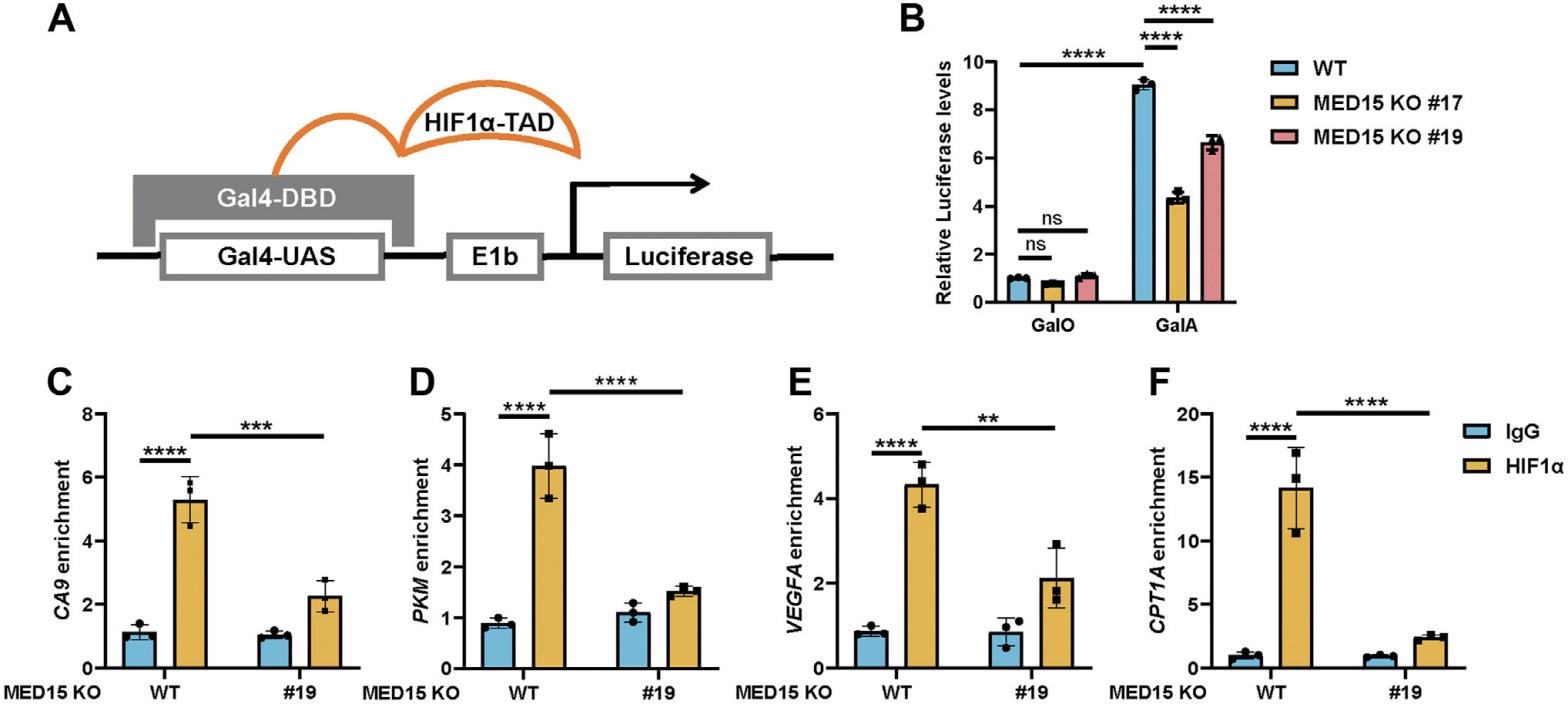
**MED15 deficiency inhibits the TAD function of HIF1α and the binding of HIF1α to HRE.***A*, schematic diagram of Gal4/UAS reporting system. The DNA-binding domain of Gal4 (Gal4-DBD) and transcriptional activation domain of HIF1α (HIF1α-TAD) are fused and expressed. *B*, luciferase reporter assays in WT or MED15 KO (KO #19) HCT116 cells. Cells were cotransfected with GalO or GalA, pSV40-Renilla, and reporter plasmid (pGal4-UAS-Elb-FLuc), followed by being exposed to 21% O_2_ or 1% O_2_ for 24 h. Data are represented as means ± SD. n = 3 biologically independent extracts. ∗∗∗∗*p* < 0.0001 by two-way ANOVA with Tukey's multiple comparisons test. *C*–*F*, Cut&Run analysis in WT or MED15 KO (KO #19) HCT116 cells. Cells were exposed to 1% O_2_ for 24 h. Cut&Run assays were performed with antibody against IgG or HIF1α followed by qPCR analysis of HRE enrichment multiples of HIF target genes *CA9* (*C*), *PKM* (*D*), *VEGFA* (*E*), and *CPT1A* (*F*). Data are represented as means ± SD. n = 3 biologically independent extracts. ∗∗*p* < 0.01, ∗∗∗*p* < 0.001, ∗∗∗∗*p* < 0.0001 by two-way ANOVA with Tukey's multiple comparisons test. CA9, carbonic anhydrase 9; CPT1A, carnitine O-palmitoyltransferase 1; E1b, a TATA box from the promoter region of adenovirus *E1b* gene; Gal4-UAS, Gal4 binding DNA sequence; HIF, hypoxia-inducible factor; MED15, Mediator complex subunit 15; ns, not significant; PKM, pyruvate kinase; VEGFA, vascular endothelial growth factor A.

Previous study has shown that MED15 physically interacts with the HIF1α transcriptional activation domain (TAD) encompassing residues 532 to 826 (24). To further explore the effect of MED15 on HIF1α TAD function, we employed a Gal4/UAS-based reporter assay (Fig. 9*A*) (48). The expression vector pGalA encoded the Gal4 DNA-binding domain fused to the HIF1α TAD (amino acids 532–826). The reporter plasmid, pGal4-UAS-Elb-FLuc, contained Gal4 DNA-binding elements (UAS) upstream of the firefly luciferase (Fluc) coding sequence. WT and MED15 KO HCT116 cells were cotransfected with pGalA or pGalO (control lacking the TAD), pGal4-UAS-Elb-FLuc, and a control reporter (pSV40-Renilla) expressing renilla luciferase. MED15 deficiency significantly impaired Gal4 transcriptional activity (Fig. 9*B*), indicating that MED15 is crucial for HIF1α TAD function. We further investigated the potential role of MED15 in HIF1α binding to target gene HRE sequences under hypoxic conditions. Cut&Run assays were performed in HCT116 cells exposed to 1% O_2_ for 24 h. Analysis of enriched DNA fragments revealed a significant reduction in hypoxia-induced HIF1α binding to the promoter of *CA9*, *PKM*, *VEGFA*, and *CPT1A* in MED15 KO cells (Fig. 9, *C*–*F*). Taken together, these data demonstrate that MED15 promotes HIF1α transcriptional activity by enhancing both HIF1α TAD function and HIF1α binding to target gene HREs.

## Discussion

The Mediator, a conserved multisubunit coregulator complex, is essential for regulating gene expression. Different subunits of the Mediator complex can interact with specific transcription factors and cofactors, thereby influencing tissue-specific gene regulation and impacting diverse biological processes (49, 50, 51, 52, 53). In this study, we uncover a previously unknown role for MED15, a subunit of the Mediator complex, in regulating HIF-mediated processes. We identify a novel regulatory axis, MED15-HIF-CPT1A, with potential implications for tumorigenesis and lipid metabolism.

Previous studies have demonstrated that MED15 interacts with transcription factors such as SREBP1 and TGFβ, thereby influencing lipid metabolism and cell proliferation, respectively (27, 29, 31, 54). Our current research extends these findings by revealing a novel role for MED15 in regulating HIF activity. MED15 deletion led to decreased HIF activity and downregulation of multiple HIF target genes, including those involved in glycolysis. Notably, MED15 modulated the activity of both HIF1α and HIF2α without affecting their protein levels, suggesting a posttranslational mechanism of regulation. Considering the primary role of the Mediator complex as a transcriptional coactivator, this regulatory function of MED15 is not unexpected. A recent study further highlights the versatility of MED15 by demonstrating its interaction with tissue-specific transcription factors NKX6-1 and NeuroD1, which are essential for β cell maturation (55).

The ability of MED15 to regulate HIF transcriptional activity was also validated in zebrafish. MED15 deficiency led to decreased HIF transcriptional activity and impaired hypoxia tolerance in zebrafish. Previous studies have shown that knockdown of MED15 using morpholinos resulted in the inhibition of the Nodal signaling pathway (56). However, in our study, we did not observe any significant abnormalities in the Nodal signaling pathway or developmental defects in MED15 KO zebrafish, which survived to adulthood. Further mechanistic studies revealed that MED15 facilitated the binding of HIF1 to HREs in the chromatin of hypoxic cancer cells. This finding is particularly intriguing when contrasted with the established role of Mediator-associated kinase CDK8. While CDK8 is required for the induction of many HIF-1 target genes, it appears dispensable for HIF chromatin binding (24). These observations suggest that different Mediator subunits may exert distinct effects on HIF1 regulation. We propose that the physical interactions between HIFs, coactivators like MED15, and components of the basal transcriptional machinery are complex and likely context-dependent. MED15 may not only facilitate initial HIF1 binding to HREs but might also stabilize this interaction through mechanisms such as regulating RNA polymerase II recruitment or mediating altered chromatin modifications. Unraveling the precise molecular mechanisms by which MED15 modulates HIF1 activity remains an important area for further investigation.

Given the established role of HIF in tumorigenesis, we hypothesized that MED15 might promote tumor growth through its regulation of HIF activity. Indeed, MED15 deletion significantly suppressed tumor growth *in vitro* and xenograft models of colorectal and renal clear cell carcinoma. This finding aligns with the broader role of MED15 in cancer, as it has been implicated in several other cancer-related processes, including modulation of TGFβ/Smad signaling, promotion of breast cancer cell metastasis, and association with poor prognosis in prostate cancer (31). In the specific context of renal cell carcinoma, MED15 has been identified as a fusion partner of TFE3 in a rare subtype, suggesting that aberrant activation of the TFE3-MED15 fusion protein may contribute to tumorigenesis (57). In addition, hypoxia induced MED15 expression in a HIF-dependent manner. Similar to other HIF target genes, such as *EPO*, *PD-L1*, and *PADI4*, the HRE site of MED15 has been identified downstream of the transcription start site (58, 59, 60). These findings suggest a positive feedback loop between HIF and MED15, potentially contributing to tumorigenesis.

**Figure 10:**
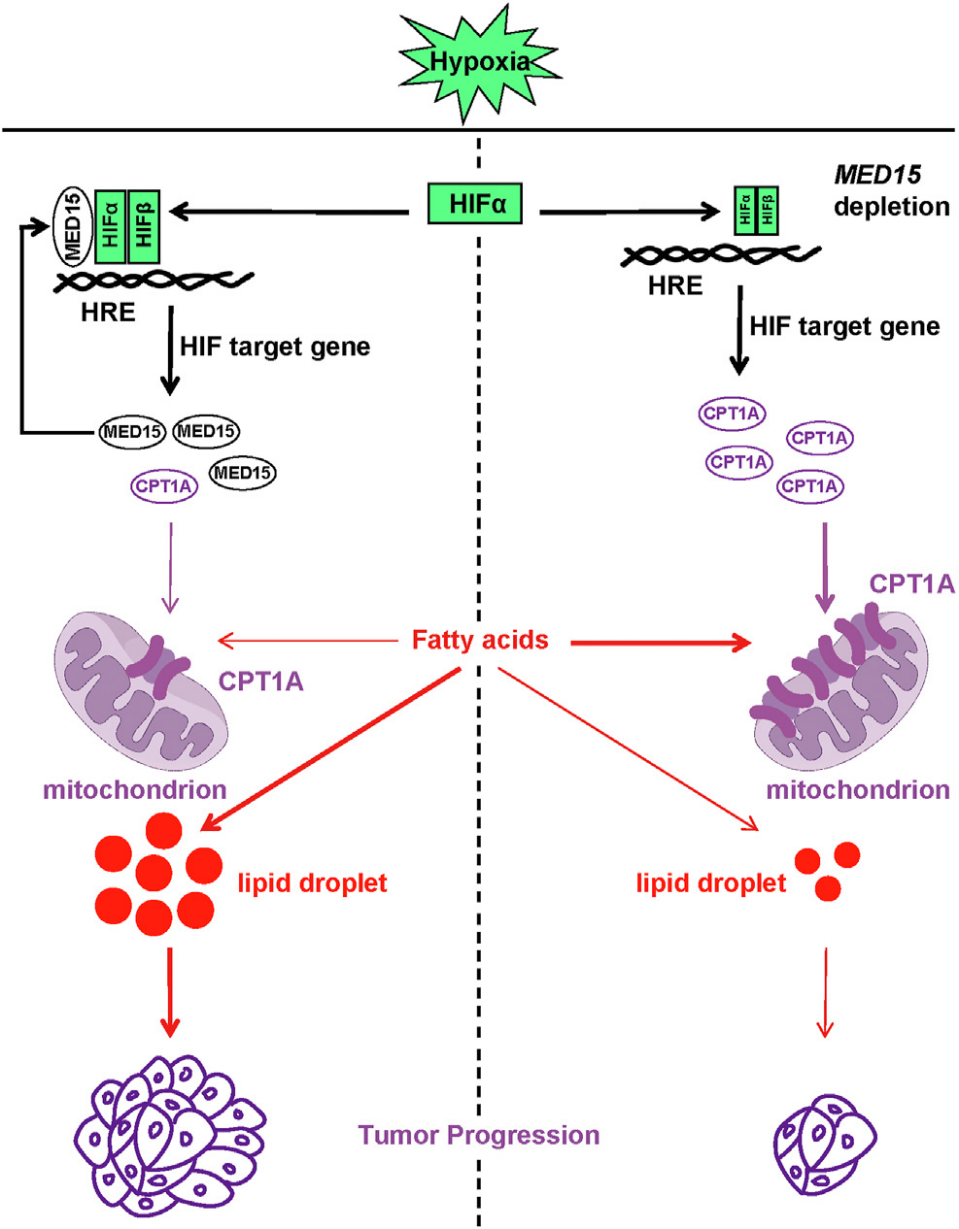
**MED15–HIF–CPT1A axis maintains lipid homeostasis and promotes tumor progression.** The proposed model suggests that MED15 promotes cancer progression during hypoxia. In hypoxia, stabilized HIFα forms a dimeric complex with HIFβ and binds to the HRE of MED15. MED15 acts as a transcriptional coactivator of HIF, facilitating its binding to the promoter of CPT1A. Due to its role as a HIF-repressed gene, CPT1A protein levels decrease, leading to increased storage of fatty acids in the cytoplasm as LD, thereby promoting tumor progression. CPT1A, carnitine O-palmitoyltransferase 1; HIF, hypoxia-inducible factor; MED15, Mediator complex subunit 15.

MED15 is known to regulate lipid metabolism. We observed a reduction in tumor cell LD upon MED15 deletion under both normoxic and hypoxic conditions. Similarly, MED15 deficiency suppressed lipid accumulation in zebrafish, suggesting a conserved role for MED15 in regulating lipid metabolism across species. MED15 is classically considered as a co-activator of SREBP1 involved in maintaining lipid homeostasis (8, 61, 62). However, the role of MED15 in hypoxia-induced lipid homeostasis is still unclear. Our data showed that pharmacological inhibition or overexpression of FASN has no significant effect on LD content in cells. Interestingly, we observed that MED15 deficiency upregulated CPT1A, a key HIF target gene and fatty acid oxidation enzyme known to be suppressed by HIF in renal clear cell carcinoma (8). We further demonstrated that CPT1A inhibition rescued the reduction in tumor cell lipid content caused by MED15 loss, suggesting a novel role for MED15 in regulating hypoxia-induced lipid metabolism through the HIF-CPT1A axis (Fig. 10). Our data indicate that fatty acid oxidation rather than *de novo* synthesis is the main pathway through which MED15 maintains hypoxia-induced LD homeostasis. Further investigation is needed to understand the precise mechanisms by which MED15 interacts with SREBP1 and HIF, as well as the potential interplay between these pathways in different cell types and disease states.

## Experimental procedures

### Reagents and antibodies

All chemicals were purchased from Sangon Biotech unless otherwise noted. CoCl_2_ (#7791-13-1) was purchased from Bio Basic Inc. CPT1A inhibitor Etomoxir (#124083-20-1), FASN inhibitor Denifanstat (#1399177-37-7), ANT inhibitor agaric acid (#666-99-9), and NADH:ubiquinone oxidoreductase (complex I) inhibitor rotenone (#83-79-4) were purchased from TargetMol. The antibodies used in this study are listed below: HIF1α (1:1000, #610959, BD Biosciences); HIF2α (1:1000, #NB100-122, Novus Biologicals); β-Actin (1:2000, #abs137975, Absin Biosciences); CA9 (1:500, #sc-365900, Santa Cruz); CPT1A (1:5000, #15184-1-AP, Proteintech); HIS (1:1000, #250114, Zen Bioscience); MED15 (1:1000, #11566-1-AP, Proteintech); MED14 (1:200, #sc-81236, Santa Cruz); MED16 (1:200, # sc-130439, Santa Cruz); MED24 (1:100, #sc-137196, Santa Cruz); HIF1β (1:200, # sc-17811, Santa Cruz); VHL (1:200, #sc-135657, Santa Cruz).

### Cell culture and hypoxia experiments

HEK293T/17, HCT116, and pVHL-mutant 786-O cell lines were obtained from ATCC and authenticated by ShCellBank. All cell lines were cultured in either Dulbecco's modified Eagle's medium or RPMI-1640 supplemented with 10% fetal bovine serum and 1% penicillin-streptomycin at 37 °C in a humidified incubator with 5% CO_2_. All cell lines were mycoplasma-free and were authenticated by short tandem repeat DNA profiling analysis. For hypoxia experiments, cells were incubated in a closed chamber containing a hypoxic gas mixture of 1% O_2_, 5% CO_2_, and 94% N_2_ as previously described (63). To assess hypoxia tolerance in adult zebrafish, an oxygen monitor and an automatic nitrogen injection system maintained a hypoxic environment within the test aquarium. Nitrogen was directly injected when oxygen levels exceeded a set threshold, as previously described (34, 64). Adult zebrafish were introduced to the aquarium at a stable oxygen concentration of 5% (1.67 ± 0.11 mg/L), and their survival was monitored by recording mortality every hour.

### Plasmids and transient transfection

Plasmids pb-3×Flag-HIF1α, pBaBe-HA-HIF2α, pcDNA3.1-MYC-HIS-MED15, pcDNA3.1-MYC-HIS-FASN, pGL2-MED15-HREs, and the expression vector pGalA (encoding a fusion of the GAL4 DNA-binding domain to the TAD (532-826) of the transcription factor HIF1α, under the control of the cytomegalovirus promoter) were generated by PCR subcloning as described previously (63, 65). Plasmids pGal4-UAS-Elb-FLuc and pGalO were purchased from the SUZHOU Bioresearch Innovation Centre. pLKO.1-GFP-MED15-sh1 and pLKO.1-GFP-MED15-sh2 were generated by inserting double-stranded oligonucleotides targeting the MED15 sequence into the pLKO.1-GFP vector. The shRNA sequences designed included MED15 shRNA-1 (5′-GATCAACAAGATCGACAAGAA-3′) and MED15 shRNA-2 (5′-TCTCGTGCCAGGCTCATTAT-3′). The plasmid pcDNA3.1-MYC-HIS-MED15 (PM), containing the MED15 point mutation, was constructed by designing primers (The specific PCR primers used are listed in Table S1) to introduce the mutation into MED15 using a targeted mutation method. The plasmid was then verified by sequencing after transformation and screening with DpnI enzyme. Transient transfection was performed using PEI.

### Lentivirus production and viral transduction

Lentivirus production involves the cotransfection of HEK293T/17 cells with the target transfection plasmid, packaging plasmid (psPAX2), and envelope plasmid (pMD2.G) using PEI. The virus-containing supernatant was collected at 48 and 72 h after transfection, filtered, and used directly for subsequent transduction. For transduction, cells were seeded at 50 to 70% confluency and then incubated with lentiviral supernatant and polybrene for 24 h. Successfully transduced cells were selected with puromycin for 5 days.

### Tumor xenograft establishment

Female BALB/c nude mice (6 weeks old) were obtained from Beijing Vital River Laboratory Animal Technology. All animal procedures were approved by the Ethical Committee of Experimental Animal Care, Ocean University of China. Human 786-O renal carcinoma cells (1 × 10^6^ cells in 100 μl RPMI-1640) and HCT116 colon carcinoma cells (2 × 10^6^ cells in 100 μl Dulbecco's modified Eagle's medium) were used to establish subcutaneous xenografts. Mice harboring 786-O xenografts were monitored for tumor development every 6 days, while those with HCT116 xenografts were monitored every 3 days. Tumor growth was assessed by caliper measurements, and tumor volume was calculated using the formula V = ½ × L × W^2^. Upon reaching a predetermined volume of approximately 2000 mm³, tumors were excised and divided for further analysis. One portion was fixed for IHC, and the remaining portion was snap-frozen in liquid nitrogen for subsequent Western blot analysis.

### Generation of *med15* deletion zebrafish

Zebrafish (*Danio rerio*) were housed in a recirculating water system (ESEN) at a constant temperature of 28 ± 0.5 °C with a 14-h light/10-h dark cycle. CRISPR/Cas9 mutagenesis was employed to generate *med15* mutant zebrafish lines. In brief, single-cell embryos were injected with a mixture of synthetic guide RNA (gRNA) targeting *med15* (5′-GGGATGCTCAGCTGAAGAGG-3′) and Cas9 protein (GenScript). Two days postinjection, genomic DNA was extracted from randomly selected embryos for PCR analysis to assess mutation efficiency. F0 generation embryos harboring verified mutations were raised to adulthood and outcrossed to WT zebrafish to establish the F1 generation. To generate F2 zebrafish, heterozygous F1 fish were crossed. The F2 zebrafish were genotyped by T7E1 mutagenesis assay and DNA sequencing. The specific genotyping PCR primers used are listed in Table S1. Stable mutant lines were maintained by inbred crossing. All animal procedures were conducted according to the guidelines established by the University Committee on the Use and Care of Animals at the Ocean University of China.

### Western blot assay

Protein expression levels were determined by Western blot as described previously (63). Briefly, mouse tumor tissue or whole-cell lysates were prepared using RIPA buffer. Lysates were resolved by SDS-PAGE on either 8% or 10% polyacrylamide gels and transferred to polyvinylidene fluoride membranes. Membranes were then blocked and incubated overnight at 4 °C with primary antibodies. Following washes, secondary antibodies were applied for 2 h at room temperature. Finally, protein bands were visualized using an enhanced chemiluminescence reagent kit (Biosharp).

### Generation of KO cell lines

#### For the generation of *MED15* KO cell lines

HCT116 and 786-O cell lines were engineered to harbor *MED15* knockouts using the CRISPR/Cas9 system. Briefly, single-guide RNA (sgRNA) sequences targeting *MED15* (5′-TTGTTGATCATGCGGCGCAG-3′) was designed using Benchling software (https://www.benchling.com/). The designed sgRNA was then ligated into the pLentiCRISPRv2 vector. These pLentiCRISPRv2-sgMED15 plasmids were cotransfected with psPAX2 and pMD2.G plasmids into HEK293T/17 cells to generate lentiviruses as previously described (63). The resulting viral supernatants were used to infect HCT116 and 786-O cells in the presence of polybrene. Following puromycin selection, KO clones were isolated from the sgRNA-targeted pools by limiting dilution in 96-well plates. These clones were subsequently verified by sequencing.

#### For the generation of *HIF1β* KO cell line

HCT116 cell lines were engineered to harbor *HIF1β* knockouts using the CRISPR/Cas9 system. Briefly, sgRNA sequences targeting *HIF1β* (5′-CAGTCCTCCGTCTCCTCACC-3′) was designed using Benchling software (https://www.benchling.com/). The designed sgRNA was then ligated into the pLentiCRISPRv2 vector. These pLentiCRISPRv2-sgHIF1β plasmids were cotransfected with psPAX2 and pMD2.G plasmids into HEK293T/17 cells to generate lentiviruses as previously described (63). The resulting viral supernatants were used to infect HCT116 cells in the presence of polybrene. Following puromycin selection, KO clones were isolated from the sgRNA-targeted pools by limiting dilution in 96-well plates. These clones were subsequently verified by sequencing.

### Luciferase reporter assay

All reporter analyses were performed in HCT116 cells. HCT116 cells were seeded into 24-well plates. For the p2.1 assay, HCT116 cells were cotransfected with p2.1 and pb-3×Flag-HIF1α or pBaBe-HA-HIF2α plasmids, or HCT116 cells were transfected with p2.1 plasmid alone and exposed to 1% O_2_ or 21% O_2_ for 24 h. For the Gal4 assay, cells were cotransfected with pGal4-UAS-Elb-FLuc and pGalA or pGalO plasmids. Transfection efficiency was estimated by cotransfection with the Renilla luciferase reporter plasmid pSV40-Renilla as previously reported (63). Firefly and Renilla luciferase activities were assayed using the Dual-Luciferase Reporter Assay Kit (UElandy). Relative luciferase activity was calculated by dividing firefly luciferase activity by Renilla luciferase activity.

### RNA extraction, reverse transcription, and RT‒qPCR

To quantify gene expression, total RNA was extracted from 3-days postfertilization zebrafish larvae or cultured cells using SparkZol (Sparkjade). Reverse transcription to cDNA was performed with M-MLV (Promega) reverse transcriptase following the manufacturer's protocol. Quantitative PCR was then carried out on the QuantStudio3 Real-Time PCR Instrument (Thermo Fisher Scientific) using primers listed in Table S1. Gene expression was normalized to β-actin and calculated using the 2^-ΔΔCt^ method.

### IHC assay

Tumor tissues were fixed overnight in 4% paraformaldehyde (PFA) (Sigma), dehydrated, embedded in paraffin, and sectioned at 5 μm thickness. Sections were then deparaffinized, rehydrated, and subjected to antigen retrieval using EDTA treatment and microwave heating. Endogenous peroxidase activity was quenched with 3% H_2_O_2_ solution. Subsequently, sections were incubated with primary antibody at 4 °C overnight. After washing with PBS, sections were incubated with a horseradish peroxidase–conjugated secondary antibody for 30 min at room temperature. Immunoreactivity was visualized using 3,3′-diaminobenzidine. Sections were counterstained with hematoxylin, dehydrated, mounted, and analyzed by microimaging.

### Nile Red staining for lipid droplet

HCT116 or 786-O cells were seeded in 12-well plates and fixed with 4% PFA for 15 min at room temperature. Following fixation, cells were stained with a working solution of Nile Red (Sigma) prepared according to the manufacturer's instructions for 15 min at room temperature in the dark. To visualize nuclei, DAPI (Beyotime Biotechnology) staining solution was applied for 10 min at room temperature. Finally, stained cells were imaged using a Leica TCS SP8 STED 3× laser confocal microscope (Leica). Nile Red staining area was measured using image-pro plus software.

### Cloning formation assay, cell cycle, and proliferation assay

To assess the colony-forming ability of HCT116 and 786-O cells, a clonogenic assay was performed. Cells were seeded at a density of 600 cells/well for HCT116 and 400 cells/well for 786-O in 6-well plates. Following incubation, the medium was removed, and colonies were washed twice with PBS. Cells were then fixed with 4% PFA and stained with 0.2% (w/v) crystal violet solution. Only colonies containing greater than 50 cells were counted and analyzed. Images of the stained colonies were captured using a microscope (M205 FCA, Leica).

For cell cycle analysis, WT and KO HCT116 and 786-O cells were harvested, resuspended in pre-cooled 70% ethanol, and fixed in ethanol for at least 8 h at −20 °C. The cells were washed and incubated in PBS containing propidium iodide and RNase (Yeasen Biotechnology) for 30 min at room temperature. The cell suspension was then filtered through a membrane and subjected to flow cytometric analysis (MoFlo XDP).

Cell proliferation was assessed using a growth curve assay. Briefly, normal growing WT or KO HCT116 and 786-O cells were trypsinized, counted, and seeded at equal densities into 24-well plates. Cell numbers were determined by counting cells from three wells every 24 h. The resulting data were used to generate growth curves using GraphPad Prism software.

### RNA-sequencing

HCT116 cells were cultured under normoxic and hypoxic conditions for 24 h. Total RNA was extracted using SparkZol (Sparkjade) and sent to BioMaker for library preparation and RNA-seq on the Illumina sequencing platform. Sequencing results were subjected to quality assessment, quantitative gene expression analysis, and differential gene expression analysis.

### CUT&RUN

Cells were harvested at a number of 1 × 10^6^, washed twice with pre-cooled PBS (containing protease inhibitors), and then permeabilized with NE1 (20 mM Hepes-KOH pH 7.9, 10 mM KCl, 1 mM MgCl_2_, 0.1% Triton X-100, 20% glycerol) on ice for 10 min. The permeabilized cells were incubated with concanavalin A magnetic beads (Beyotime Biotechnology) in binding buffer. After binding to the beads, cell-specific primary and secondary antibodies were added and incubated at 4 °C for 2 h. The cells were washed three times with wash buffer and incubated with pAG-MNase (Beyotime Biotechnology) for 1 h at 4 °C. The cells were washed three times with wash buffer, CaCl_2_ was added, and DNA cleavage was initiated by incubation at 0 °C for 1 h. EDTA was added to stop the reaction. DNA was extracted using 10% SDS, proteinase K, RNase A, and NaCl, then purified through phenol-chloroform-isoamyl alcohol and ethanol precipitation, and analyzed for binding sites by quantitative PCR using primers listed in Table S2.

### Whole-mount ORO staining

At 5 days postfertilization, zebrafish larvae were exposed to a high-fat diet (HFD). An HFD solution was prepared using egg yolk (30 mg) in 30 ml of embryo rearing solution. The larvae were exposed to the embryo rearing solution containing HFD for 5 days. Zebrafish larvae were collected in 1.5 ml eppendorf tubes and fixed with 4% PFA at 4 °C overnight. They were washed three times with PBS, infiltrated with 60% propylene glycol for 30 min, and prepared with 60% isopropanol in 0.3% (w/v) ORO staining solution, which was filtered after being kept away from light for 20 min at room temperature. The larvae were stained with ORO for 3 h in the dark and then washed with 60% propylene glycol to reduce background color. Finally, the larvae were washed thoroughly with PBS, and the lipid droplets in the liver were observed and photographed using a stereomicroscope (M205 FCA, Leica). All images from different groups were analyzed for comparison using the same parameters (exposure time, ISO, and aperture). The extent of ORO-positive staining in zebrafish was quantified using Image J software.

### Bioinformatics analysis

KIPAN, COADREAD, LIHC, LUAD, PRAD, STAD, BRCA, and HNSC clinical samples were downloaded from the University of California, Santa Cruz (UCSC) Xena (https://xenabrowser.net). The RNA-Seq dataset of these samples included expression levels of *MED15* and the HIF1α target genes *SREBF1*, *LRP10*, *PDGFB*, *ENO1*, *MMP2*, *MMP9*, *GAPDH*, *VEGFA*, and *HK2*. The RNA-Seq count data were normalized to transcripts per million. Patients were divided into MED15 high and MED15 low groups based on MED15 expression to analyze the association between MED15 expression levels and the expression levels of HIF target genes. Data on the expression levels of MED15 and HIF1 in ACC, CHOL, ESCA, LIHCA, and THYM were obtained from TCGA (https://www.cancer.gov/ccg/research/genome-sequencing/tcga). Expression data were log_2_(FPKM + 1) transformed. Correlation analysis between MED15 and HIF1α gene expression was performed by Gene Expression Profiling Interactive Analysis (http://gepia.cancer-pku.cn). The difference in MED15 expression levels was analyzed by GEPIA in KIRC tissue samples from TCGA and normal kidney tissue samples from Genotype-Tissue Expression Project (https://www.genome.gov/Funded-Programs). To assess the impact of MED15 on survival, we used the Kaplan-Meier plotter (http://www.kmplot.com/analysis/), which included 76 KIRC patient samples with low MED15 and 521 KIRC patient samples with high MED15. Expression data were transformed by log_2_(FPKM + 1). Unless otherwise stated, all data were normalized to Fragments Per Kilobase of Exon Model per Million mapped fragments (FPKM), and R software was used for statistical analysis.

### Statistical analysis

Statistical analysis of the data was performed using GraphPad Prism 8.0 software (GraphPad Software, Inc), and the data are presented as mean ± SD. An unpaired Student's *t* test was used for significance comparison between two groups. Comparisons among three or more groups were performed using one-way or two-way ANOVA. A *p*-value of <0.05 was considered significant. ∗ indicates *p* < 0.05, ∗∗ indicates *p* < 0.01, ∗∗∗ indicates *p* < 0.001, ∗∗∗∗ indicates *p* < 0.0001, and "ns" indicates no significant difference.

## Data availability

All data are contained within the article.

## Supporting information

This article contains supporting information.

## Conflict of interest

The authors declare that they have no conflicts of interest with the contents of this article.

## Figures and Tables

**Supplemental Figure 14:**