# Supplementary material for: The mediator subunit complex protein MED15 promotes lipid deposition and cancer progression during hypoxia
Source: J Biol Chem. 2025 Feb 11;301(3):108296. doi: 10.1016/j.jbc.2025.108296 (PMC11930138; doi:10.1016/j.jbc.2025.108296)
Supplement: Supporting Information [file mmc1.docx]

**Supporting Information Text**

Supplemental Figure 1. MED15 promotes HIF target genes expression in cancer samples. (A-F) Analysis of mRNA levels of HIF target genes in clinical cancer samples from the TCGA cohorts. ns, not significant, **p*<0.05, ****p*<0.001, *****p*<0.0001 by Student’s *t* test. MED15, Mediator complex subunit 15; HIF, hypoxia-inducible factor; LIHC, liver hepatocellular carcinoma; LUAD, lung adenocarcinoma; PRAD, prostate adenocarcinoma; STAD, stomach adenocarcinoma; BRCA, breast invasive carcinoma; HNSC, head and Neck squamous cell carcinoma.

Supplemental Figure 2. Generation of MED15 knockout cells. (A) Sequences and site of sgRNA and PAM for constructing MED15-deficient cell lines using CRISPR/Cas9 system. (B and C) Western blot validated the knockout efficiency of MED15 in HCT116 cells (B) and 786-O cells (C). (D) Western blot of VHL in HCT116 and 786-O cells. MED15, Mediator complex subunit 15; HIF, hypoxia-inducible factor.

Supplemental Figure 3. Overexpression of MED15-PM can rescue the decrease of HIF transcriptional activity caused by knocking out MED15. Wild-type (WT) and MED15 knockout (KO #19) were co-transfected p2.1, pSV40-Renilla, and empty vector or MED15 with point mutation (MED15-PM), followed by being exposed to either 21% O_2_ or 1% O_2_ for 24 hours. Relative luciferase levels (Fluc/Rluc) were detected. Data are represented as means ± SD. n = 3 biologically independent extracts. *****p*<0.0001 by two-way ANOVA with Tukey’s multiple comparisons test. MED15, Mediator complex subunit 15.

Supplemental Figure 4. Knocking down MED15 suppressed the induction of hypoxia-response genes. (A-C) Luciferase reporter assays in HCT116 cells exposed to 21% O_2_ or 1% O_2_ for 24 hours (A) or overexpressing HIF1α (B) or HIF2α (C). (D) RT-qPCR analysis of *CA9*, *LDHA*, and *REDD1* in control or MED15 knockdown (KD1 and KD2) HCT116 cells exposed to 21% O_2_ or 1% O_2_ for 24 hours. Data are represented as means ± SD. n = 3 biologically independent extracts. **p*<0.05, ****p*<0.001, *****p*<0.0001 by two-way ANOVA with Tukey’s multiple comparisons test. (E) Western blot of HIF1α, HIF2α and CA9 in HCT116 control (Con) or MED15 knockdown (KD1 and KD2) cells exposed to 21% O_2_ or 1% O_2_ for 24 hours. MED15, Mediator complex subunit 15; *CA9*, carbonic anhydrase 9; *LDHA*, lactate dehydrogenase A; *REDD1*, protein regulated in development and DNA damage response 1; HIF, hypoxia-inducible factor.

Supplemental Figure 5. MED15 is evolutionarily conserved in human and zebrafish. Alignment of MED15 amino acid sequences from human and zebrafish, and the consensus sequence is shown. MED15, Mediator complex subunit 15.

Supplemental Figure 6. MED15 is highly expressed in renal clear cell carcinoma and predicts poor survival probabilities. (A) Representative IHC staining of MED15 in normal renal tissue and renal cancer from The Human Protein Atlas. (B) Analysis of *MED15* mRNA levels in normal tissues and tumor tissues. *****p*<0.0001 by Student’s *t* test. (C) Kaplan-Meier curves analysis of patients with renal clear cell carcinoma. Patients were divided by expression levels of *MED15* mRNA. MED15, Mediator complex subunit 15.

Supplemental Figure 7. Zebrafish larvae with *med15* mutation exhibit lipid metabolism disorders. (A-C) Representative images of Oil red O (ORO) staining for neutral lipids in 3 dpf and 5 dpf wild-type (wt) and *med15* mutant (*med15^ouc1^*) zebrafish larvae (A). Quantification of ORO staining intensity by integral optical density (IOD) in 3 dpf and 5 dpf wt and *med15^ouc1^* larvae (B). Body length of 3 dpf and 5 dpf wt and *med15^ouc1^* larvae (C). Data are represented as means ± SD. n = 30 (wt); 30 (*med15^ouc1^*). ****p*<0.001, *****p*<0.0001 by Student’s *t* test. (D-F) Representative images of ORO staining of 10 dpf wt and *med15^ouc1^* zebrafish larvae fed a five-days egg yolk with indicated concentration (D). Quantification of ORO staining intensity by IOD in 10 dpf wt and *med15^ouc1^* larvae fed different concentrationsof yolk (E). Body length of 10 dpf wt and *med15^ouc1^* larvae fed different concentrations of yolk (F). Data are represented as means ± SD. n = 22 (wt); 22 (*med15^ouc1^*). **p*<0.05, *****p*<0.0001 by two-way ANOVA with Tukey’s multiple comparisons test. The blue rectangle shows the ORO staining area on the head, the yellow coil represents the ORO staining area in the internodal blood vessels, while the green line surrounds the ORO staining areas in the arteries and veins. *med15*, Mediator complex subunit 15.

Supplemental Figure 8. The expression levels of some lipid metabolism related genes remain unchanged after knocking out MED15. (A) RT-qPCR analyzed the mRNA levels of lipid metabolism related proteins in wild-type (WT) and MED15 KO (#19) HCT116 cells treated under 21% O_2_ or 1% O_2_ for 72 hours. Data are represented as means ± SD. n = 3 biologically independent extracts. ns, not significant, **p*<0.05, ***p*<0.01, ****p*<0.001, *****p*<0.0001 by two-way ANOVA with Tukey’s multiple comparisons test. (B) RT-qPCR analysis of the mRNA levels of lipid metabolism related genes in wild-type (WT) and MED15 KO (#5) 786-O cells. Data are represented as means ± SD. n = 3 biologically independent extracts. ns, not significant by Student’s *t* test. *FABP3*, fatty acid-binding protein; *ACACA*, acetyl-CoA carboxylase 1; *HIG2*, hypoxia-inducible lipid droplet-associated protein 2; *LRP1*, prolow-density lipoprotein receptor-related protein 1; *PPARα*, peroxisome proliferator-activated receptor α; *SREBP1*, sterol regulatory element-binding protein 1; *VLDLR*, very low-density lipoprotein receptor; *AGPAT2*, 1-acyl-sn-glycerol-3-phosphate acyltransferase β; *MCAD*, medium-chain specific acyl-CoA dehydrogenase; *GLS1*, glutaminase 1; *ATGL* patatin-like phospholipase domain-containing protein 2; MED15, Mediator complex subunit 15.

Supplemental Figure 9. FASN inhibitor cannot impair hypoxia dependent lipid droplet accumulation. (A and B) Nile Red and DAPI staining assay of HCT116 cells exposed to 21% O_2_ or 1% O_2_ for 72 hours in the presence of DMSO or Denifanstat (25 μM). Representative images from three independent experiments are shown in (A). Quantification of Nile Red staining area is shown in (B). Data are represented as means ± SD. n = 21 (21% O_2_ WT DMSO); 21 (21% O_2_ WT Denifanstat); 21 (1% O_2_ WT DMSO); 21 (1% O_2_ WT Denifanstat). ns, not significant, *****p*<0.0001 by two-way ANOVA with Tukey’s multiple comparisons test. (C and D) Nile Red and DAPI staining assay of 786-O cells treated with DMSO or Denifanstat (25 μM) for 72 hours. Representative images from three independent experiments are shown in (C). Quantification of Nile Red staining area is shown in (D). Data are represented as means ± SD. n = 27 (DMSO); 25 (Denifanstat). ns, not significant by Student’s *t* test.

Supplemental Figure 10. Overexpression of FASN cannot rescue lipid droplet reduction caused by knocking out MED15. (A and B) Nile Red and DAPI staining assay of WT and MED15 KO (#19) HCT116 cells transfected with EV or FASN and exposed to 21% O_2_ or 1% O_2_ for 72 hours. Representative images from three independent experiments are shown in (A). Quantification of Nile Red staining area is shown in (B). Data are represented as means ± SD. n = 25 (21% O_2_ WT EV); 25 (21% O_2_ WT FASN); 25 (21% O_2_ MED15 KO #19 EV); 25 (21% O_2_ MED15 KO #19 FASN); 25 (1% O_2_ WT EV); 25 (1% O_2_ WT FASN); 25 (1% O_2_ MED15 KO #19 EV); 25 (1% O_2_ MED15 KO #19 FASN). ns, not significant, ***p*<0.01, *****p*<0.0001 by two-way ANOVA with Tukey’s multiple comparisons test. EV, empty vector; *FASN*, fatty acid synthase; MED15, Mediator complex subunit 15.

Supplemental Figure 11. Hypoxia-induced downregulation of CPT1A expression depends on HIFs. (A and B) Western blot assay of wild-type and HIF1β knockout HCT116 cells exposed to 21% O_2_ or 1% O_2_ for 72 hours (A). Relative intensities of CPT1A were quantified (D). Data are represented as means ± SD. n = 3 biologically independent extracts. ns, not significant, ***p*<0.01 by Student’s *t* test. HIF, hypoxia-inducible factor; CPT1A, carnitine palmitoyltransferase 1A.

Supplemental Figure 12. Inhibiting CPT1A can rescue MED15 deficiency dependent lipid droplet reduction. (A and B) Wild-type (WT) and MED15 knockout HCT116 cells were exposed to 21% O_2_ or 1% O_2_ for 72 hours in the presence of DMSO or Etomoxir (40 μM). LD and nuclei were staining with Nile Red and DAPI. Representative images from three independent experiments are shown in (A). Quantification of Nile Red staining area is shown in (B). Data are represented as means ± SD. n = 16 (21% O_2_ WT DMSO); 16 (21% O_2_ WT Etomoxir); 16 (21% O_2_ MED15 KO #19 DMSO); 16 (21% O_2_ MED15 KO #19 Etomoxir); 16 (1% O_2_ WT DMSO); 16 (1% O_2_ WT Etomoxir); 16 (1% O_2_ MED15 KO #19 DMSO); 16 (1% O_2_ MED15 KO #19 Etomoxir). **p*<0.05, ****p*<0.001, *****p*<0.0001 by two-way ANOVA with Tukey’s multiple comparisons test. (C and D) Nile Red and DAPI staining of wild-type (WT) and MED15 knockout 786-O treated with DMSO or Etomoxir (40 μM) for 72 hours. Representative images from three independent experiments are shown in (C). Quantification of Nile Red staining area is shown in (D). Data are represented as means ± SD. n = 13 (WT DMSO); 13 (MED15 KO #5 DMSO); 13 (WT Etomoxir); 13 (MED15 KO #5 Etomoxir); ns, not significant, ****p*<0.001, *****p*<0.0001 by two-way ANOVA with Tukey’s multiple comparisons test. MED15, Mediator complex subunit 15.

Supplemental Figure 13. Complex Ⅰ inhibitor cannot impair hypoxia dependent lipid droplet accumulation. (A and B) Nile Red and DAPI staining assay of 786-O cells treated with DMSO or Rotenone (100 nM) for 72 hours. Representative images from three independent experiments are shown in (A). Quantification of Nile Red staining area is shown in (B). Data are represented as means ± SD. n = 28 (DMSO); 30 (Rotenone). *****p*<0.0001 by Student’s *t* test. (C and D) Nile Red and DAPI staining assay of HCT116 cells exposed to 21% O_2_ or 1% O_2_ for 72 hours in the presence of DMSO or Rotenone (100 nM). Representative images from three independent experiments are shown in (C). Quantification of Nile Red staining area is shown in (D). Data are represented as means ± SD. n = 21 (21% O_2_ WT DMSO); 20 (21% O_2_ WT Rotenone); 19 (1% O_2_ WT DMSO); 23 (1% O_2_ WT Rotenone). ns, not significant, **p*<0.05, *****p*<0.0001 by two-way ANOVA with Tukey’s multiple comparisons test.

Supplemental Figure 14. ANT inhibitor do not affect lipid droplet content in cells. (A and B) Nile Red and DAPI staining assay of 786-O cells treated with DMSO or Agaric acid (20 μM) for 72 hours. Representative images from three independent experiments are shown in (A). Quantification of Nile Red staining area is shown in (B). Data are represented as means ± SD. n = 31 (DMSO); 41 (Agaric acid). ns, not significant by Student’s *t* test.
