# Supplementary material for: The mediator subunit complex protein MED15 promotes lipid deposition and cancer progression during hypoxia
Source: J Biol Chem. 2025 Feb 11;301(3):108296. doi: 10.1016/j.jbc.2025.108296 (PMC11930138; doi:10.1016/j.jbc.2025.108296)
Supplement: Supporting Information Table [file mmc2.docx]

| Supplemental Table 1. Oligonucleotide sequence used for PCR primers | |
| --- | --- |
| Primer Name | Sequence |
| Human *FASN*-F | 5’-GTTTAAACGGGCCCTATGGAGGAGGTGGTGATTGCCG-3’ |
| Human *FASN*-R | 5’-AGTCCAGTGTGGTGGGCCCTCCCGCACGCTCACGCGT-3’ |
| Human *MED15*-PM-F | 5’-CTACGCCGCATGATCAACAAGATCGACAAGAA-3’ |
| Human *MED15*-PM-R | 5’-TTGATCATGCGGCGTAGGGGCTCGATGTACTTCGAC-3’ |
| Human *ACTIN*-RT-F | 5’-AGAGCTACGAGCTGCCTGAC-3’ |
| Human *ACTIN*-RT-R | 5’-AGCACTGTGTTGGCGTACAG-3’ |
| Human *MED15*-RT-F | 5’-AGAACTTCAGTGTCCCCTCA-3’ |
| Human *MED15*-RT-R | 5’-GTACTTCGACAGCTGCTTCA-3’ |
| Human *CA9*-RT-F | 5’-GCCGCCTTTCTGGAGGA-3’ |
| Human *CA9*-RT-R | 5’-TCTTCCAAGCGAGACAGCAA-3’ |
| Human *LDHA*-RT-F | 5’-GGTTGGTGCTGTTGCATGG-3’ |
| Human *LDHA*-RT-R | 5’-TGCCCCAGCCGTGATAATGA-3’ |
| Human *REDD1*-RT-F | 5’-AGCCAGTTGGTAAGCCAGG-3’ |
| Human *REDD1*-RT-R | 5’-GCCAGAGTCGTGAGTCCAG-3’ |
| Human *CPT1A*-RT-F | 5’-ATCAATCGGACTCTGGAAACGG-3’ |
| Human *CPT1A*-RT-R | 5’-TCAGGGAGTAGCGCATGGT-3’ |
| Human *FASN*-RT-F | 5’-CGCGTGGCCGGCTACTCCTAC-3’ |
| Human *FASN*-RT-R | 5’-CGGCTGCCACACGCTCCTCT-3’ |
| Human *HMGCS1*-RT-F | 5’-CATTAGACCGCTGCTATTCTGTC-3’ |
| Human *HMGCS1*-RT-R | 5’-TTCAGCAACATCCGAGCTAGA-3’ |
| Human *HMGCR*-RT-F | 5’-TGATTGACCTTTCCAGAGCAAG-3’ |
| Human *HMGCR*-RT-R | 5’-CTAAAATTGCCATTCCACGAGC-3’ |
| Human *MCAD*-RT-F | 5’-GGAAGGAGATACCCCAGGAAT-3’ |
| Human *MCAD*-RT-R | 5’-AGCTCCGTCACCAATTAAAACAT-3’ |
| Human *LIPIN1*-RT-F | 5’-TTTCCACGTCCGCTTTGGG-3’ |
| Human *LIPIN1*-RT-R | 5’-GTGGCCAGGTGCATAGGG-3’ |
| Human *GLS1*-RT-F | 5’-GCGACACTGGCTAATGGTGG-3’ |
| Human *GLS1*-RT-R | 5’-TTGCAGGAAGACCAACATGGA-3’ |
| Human *ATGL*-RT-F | 5’-CCCACTTCAACTCCAAGGAC-3’ |
| Human *ATGL*-RT-R | 5’-TAGAGTGGCAGGTTGTCTGA-3’ |
| Human *FABP3*-RT-F | 5’-GTGGAGTTCGATGAGACAACAGC-3’ |
| Human *FABP3*-RT-R | 5’-TGGTCTCTTGCCCGTCCCATTT-3’ |
| Human *ACACA*-RT-F | 5’-ATGTCTGGCTTGCACCTAGTA-3’ |
| Human *ACACA*-RT-R | 5’-CCCCAAAGCGAGTAACAAATTCT-3’ |
| Human *HIG2*-RT-F | 5’-ACGAGGGCGCTTTTGTCTC-3’ |
| Human *HIG2*-RT-R | 5’-AGCACAGCATACACCAGACC-3’ |
| Human *LRP1*-RT-F | 5’-AGTCGTCTCTGCAGACTTGC-3’ |
| Human *LRP1*-RT-R | 5’-TGCGTTCTTGAAGGAGCCAT-3’ |
| Human *PPARα*-RT-F | 5’-TGTAGAATCTGCGGGGACAAG-3’ |
| Human *PPARα*-RT-R | 5’-GGTCGCACTTGTCATACACCA-3’ |
| Human *SREBP1*-RT-F | 5’-ACTTCTGGAGGCATCGCAAGCA-3’ |
| Human *SREBP1*-RT-R | 5’-AGGTTCCAGAGGAGGCTACAAG-3’ |
| Human *VLDLR*-RT-F | 5’-GAAGATGAAGAAAACTGTGGCAA-3’ |
| Human *VLDLR*-RT-R | 5’-GCACAGTCCAGCTCATCACT-3’ |
| Human *AGPAT2*-RT-F | 5’-GTGGGCCTCATCATGTACCTC-3’ |
| Human *AGPAT2*-RT-R | 5’-ACGATGGGCACGTTCTCCC-3’ |
| Human MED15-HRE1-F | 5’-CATGTATGTATATATACGTATTTACGTGTATATATGTATGCATACATATAC-3’ |
| Human MED15-HRE1-R | 5’-TCGAGTATATGTATGCATACATATATACACGTAAATACGTATATATACATACATGGTAC-3’ |
| Human MED15-HRE2-F | 5’-CGGGAGGCTGAGGCAGGAGAATGGCGTGAACCCAGGAGGCGGAGCTTGCAC-3’ |
| Human MED15-HRE2-R | 5’-TCGAGTGCAAGCTCCGCCTCCTGGGTTCACGCCATTCTCCTGCCTCAGCCTCCCGGTAC-3’ |
| Human MED15-HRE3-F | 5’-CAAAAAATTAGCCAAGTATGGTGGCGTGCGCCTGTAATCCCAGTTACCTGC-3’ |
| Human MED15-HRE3-R | 5’-TCGAGCAGGTAACTGGGATTACAGGCGCACGCCACCATACTTGGCTAATTTTTTGGTAC-3’ |
| Human MED15-HRE4-F | 5’-CGGCTGAGTTCTCCATGAAGGGGACGTGGCTGCTGGTCAAAAGGGAAAGCC-3’ |
| Human MED15-HRE4-R | 5’-TCGAGGCTTTCCCTTTTGACCAGCAGCCACGTCCCCTTCATGGAGAACTCAGCCGGTAC-3’ |
| Human MED15-HRE5-F | 5’-CATGTCTAAAAATTAATTAACGTGGTTTAGGGGCCTCACATTCCCAACCCC-3’ |
| Human MED15-HRE5-R | 5’-TCGAGGGGTTGGGAATGTGAGGCCCCTAAACCACGTTAATTAATTTTTAGACATGGTAC-3’ |
| Human MED15-HRE6-F | 5’-CAGGTCTGCTGCGGGAGCACCTGCCGTGTCCGACAGCTGCACTGGAAGAGC-3’ |
| Human MED15-HRE6-R | 5’-TCGAGCTCTTCCAGTGCAGCTGTCGGACACGGCAGGTGCTCCCGCAGCAGACCTGGTAC-3’ |
| Human MED15-HRE7-F | 5’-CCGACAGCTGCACTGGAAGAGGGGCGTGTGTCTCCCAGCCGGGCGGAACCC-3’ |
| Human MED15-HRE7-R | 5’-TCGAGGGTTCCGCCCGGCTGGGAGACACACGCCCCTCTTCCAGTGCAGCTGTCGGGTAC-3’ |
| Human MED15-HRE8-F | 5’-TAACATAACCCGGGAGGTACCTAGGGTGGGGCAGGAACAA |
| Human MED15-HRE8-R | 5’-AATTGAGATGCAGATCTCGAGGTGTCTCAGGCAAATCTGTGGC |
| Human MED15-3000~+1-F | 5’-TAACCCGGGAGGTACGGGAGGGTTGTCCTAAGTATCC |
| Human MED15-3000~+1-R | 5’-ATTGAGATGCAGATCCAGGCCCAAACCCGGAAGCCGT |
| Human MED15-HRE8-MUT-F | 5’-AAAAACAAGTCACAGGGGATGCGATGGCCTGGC |
| Human MED15-HRE8-MUT-R | 5’-CCCCTGTGACTTGTTTTTATACATCCAGATGGCCTGAAGTAA |
| Zebrafish *actin*-RT-F | 5’-AGGTCATCACCATTGGCAAT-3’ |
| Zebrafish *actin-*RT-R | 5’-GATGTCGACGTCACACTTCAT-3’ |
| Zebrafish *med15*-RT-F | 5’-ACTTCAAGGTGAAGTGGCTCGG-3’ |
| Zebrafish *med15*-RT-R | 5’-GGGATGCTCAGCTGAAGAGGG-3’ |
| Zebrafish *cited2*-RT-F | 5’GTTCCGAGACAGTATCGCTAAG--3’ |
| Zebrafish *cited2*-RT-R | 5’-ATCAAGACCTCCTCGTCAATAA-3’ |
| Zebrafish *phd3*-RT-F | 5’-CGCTGCGTCACCTGTATT-3’ |
| Zebrafish *phd3*-RT-R | 5’-GCATACGACGGCTGAACT-3’ |
| Zebrafish *runx1*-RT-F | 5’-GGGACGCCAAATACGAACCT-3’ |
| Zebrafish *runx1*-RT-R | 5’-GCAGGACGGAGCAGAGGAAG-3’ |
| Zebrafish *redd1*-RT-F | 5’-TGGACTCTGACTCCGACAACC-3’ |
| Zebrafish *redd1*-RT-R | 5’-ACCACTTCTTTACACAACGCCTC-3’ |
| Zebrafish *gadd34*-RT-F | 5’-GAAGAGCAGTGGGAAGAAGG-3’ |
| Zebrafish *gadd34*-RT-R | 5’-CTGAACTCTCCTCCTGAAACG-3’ |
| Zebrafish *epo*-RT-F | 5’-AGGAGGCAGGATATGGACTATTAC-3’ |
| Zebrafish *epo*-RT-R | 5’-ACAGTTGGAGGTGCTTGAGG-3’ |
| Zebrafish *med15*-genotyping-F | 5’- TCTGAAACTGAGCCCACTAT -3’ |
| Zebrafish *med15*-genotyping-R | 5’- GACCAATCAGTGACCAACAG -3’ |

| Supplemental Table 2. Oligonucleotide sequence used for CUT&RUN -qPCR primers | |
| --- | --- |
| Primer Name | Sequence |
| Human *CPT1A*-F | 5’-ACCCAGCTGGTCCTCTTTTA-3’ |
| Human *CPT1A*-R | 5’-ACAAACATGAGCCACTGCAC-3’ |
| Human *CA9*-F | 5’-TCTCGTTTCCAATGCACGTACAGC-3’ |
| Human *CA9*-R | 5’-AGTGACAGCAGCAGTTGCACAGT-3’ |
| Human *VEGFA*-F | 5’-CCTCAGTTCCCTGGCAACATCTG-3’ |
| Human *VEGFA*-R | 5’-GAAGAATTTGGCACCAAGTTTGT-3’ |
| Human *PKM*-F | 5’-TTCCTGCCTCTTGGTATGAC-3’ |
| Human *PKM*-R | 5’-CGGCTTGTTCCCTCCTAC-3’ |
